# Emerging Biomedical Applications of Nano-Chitins and Nano-Chitosans Obtained via Advanced Eco-Friendly Technologies from Marine Resources

**DOI:** 10.3390/md12115468

**Published:** 2014-11-19

**Authors:** Riccardo A. A. Muzzarelli, Mohamad El Mehtedi, Monica Mattioli-Belmonte

**Affiliations:** 1 Faculty of Medicine, Department of Clinical & Molecular Sciences, Polytechnic University of Marche, IT-60100 Ancona, Italy; E-Mail: m.mattioli@univpm.it; 2 Faculty of Engineering, Department of Industrial Engineering & Mathematical Sciences, Polytechnic University of Marche, IT-60100 Ancona, Italy; E-Mail: elmehtedi@univpm.it

**Keywords:** chitin, chitosan, electrospinning, nanofibrils, biomedical uses

## Abstract

The present review article is intended to direct attention to the technological advances made in the 2010–2014 quinquennium for the isolation and manufacture of nanofibrillar chitin and chitosan. Otherwise called nanocrystals or whiskers, n-chitin and n-chitosan are obtained either by mechanical chitin disassembly and fibrillation optionally assisted by sonication, or by e-spinning of solutions of polysaccharides often accompanied by poly(ethylene oxide) or poly(caprolactone). The biomedical areas where n-chitin may find applications include hemostasis and wound healing, regeneration of tissues such as joints and bones, cell culture, antimicrobial agents, and dermal protection. The biomedical applications of n-chitosan include epithelial tissue regeneration, bone and dental tissue regeneration, as well as protection against bacteria, fungi and viruses. It has been found that the nano size enhances the performances of chitins and chitosans in all cases considered, with no exceptions. Biotechnological approaches will boost the applications of the said safe, eco-friendly and benign nanomaterials not only in these fields, but also for biosensors and in targeted drug delivery areas.

## 1. Introduction and Scope

Discovered over two centuries ago, chitin plays today protagonist roles in innovative research. A number of current research works are inspired by the natural chitin structure: the occurrence of chitin in living organisms since the Cambrian life explosion is explained by the recognition of its very good performance and versatility in perception, protection, aggression, feeding, reproduction and fertilization, locomotion and flight [1,2,3,4,5,6,7].

Chitin nanofibers are mainly prepared from crustacean and diatomaceous chitin powders according to newly adopted approaches and protocols. In particular, recent articles deal with the following advances: (I) n-chitin isolated after hydrolysis in diluted HCl; (II) n-chitin isolated mechanically in the presence of minor amounts of acetic acid; (III) n-chitosan obtained from partially deacetylated chitin; (IV) mechanical fibrillation; (V) fibrillation with the aid of sonication; (VI) manufacture of chitin nanofibers by e-spinning; (VII) preparation and spinning of chitin solutions in ionic liquids; (VIII) manufacture of aerogels. These advances are of such importance that they overshadow the technology developed during the previous years, as it will be apparent below.

In his review, Araki [8] introduced recent results on electrostatic and steric stabilizations of nanofibrils, together with brief and basic descriptions of their stabilization mechanisms. Chitin nanofibers were isolated from the cell walls of five types of mushrooms by the removal of glucans, minerals, and proteins, followed by a simple grinding treatment under acidic conditions. The width of the nanofibers depended on the type of mushrooms and varied in the range 20–28 nm; the crystalline structure was maintained and glucans remained on the nanofiber surface [9]. By similar means, chitin nanofibers (Φ 10–20 nm) were isolated from prawn shells under mild conditions [10].

Once isolated, chitin nanofibers can be derivatized to modify their surfaces permanently: for example they are fully acetylated with acetic anhydride in warm anhydrous pyridine. The acetylation can be controlled by changing the reaction time; it proceeds heterogeneously from the surface to the core [11]. As an example of elaborated application, covalent coupling of chitin nanofibrils with magnetic nanoparticles in aqueous media was proposed for DNA extraction [12].

The e-spun composite nanofibers are of great significance owing to their large surface area to volume ratio, besides porosity, stability, and permeability. The functionality and applicability of these nanostructures were further improved by incorporating secondary phases that include magnetic metal oxides, carbon nanotubes, precious metals, and hydroxyapatite. Nanofibrous materials compatible with the extracellular matrix and capable to promote the adhesion of cells are being developed as engineered scaffolds for the skin, heart, cornea, nerves, bone, blood vessels, and other tissues. The review article by Sahay *et al.* [13] discusses the applicability of these composite fibers in energy, filters, biotechnology, sensors, packaging materials, and indicates technological issues, research challenges, and emerging trends. The reader is referred to fundamental works [14,15,16,17,18,19,20,21,22,23,24,25,26,27] for complementary information.

### 1.1. β-Chitin: The Simplest 2D Hydrogen Bonded Polymorph

**Figure 1:**
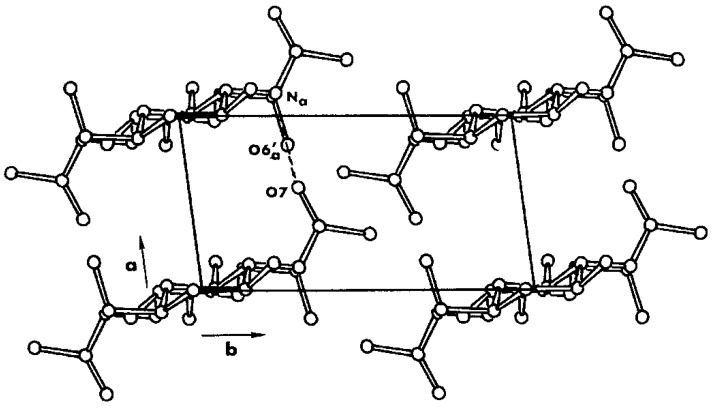
Projections of the structure of β-chitin: no linkage exists along the b axis. The polymer can be easily fibrillated by acting on this point of weakness. Courtesy of Riccardo A. A. Muzzarelli.

β-Chitin is found in association with proteins in squid pens: the dry pen contains 31% chitin whose viscosity average MW is over 2 MDa, and the crystallinity index is 75%. The degree of acetylation was found to be 0.96 [28]. The content of inorganic compounds is very low. The crystal structure of β-chitin [29,30,31,32,33] lacks hydrogen bonds along the b axis (Figure 1), and therefore it is more susceptible than α-chitin to intra-crystalline swelling, acid hydrolysis even at low acid concentrations, and loss of scarcely crystalline fractions. In the squid gladius, the chitin molecules are known to form nano-crystallites of monoclinic lattice symmetry wrapped in a protein layer, resulting in β-chitin nanofibrils [34]. Three β-chitin structures (anhydrous, dihydrate, and mono-ethylenediamine) were recently determined by synchrotron X-ray and neutron fiber diffraction [35]. The optimal deproteination and demineralisation conditions were defined by Youn *et al.* [36].

### 1.2. α-Chitin: The 3D Hydrogen Bonded Polymorph

α-Chitin is the most abundant polymorph; it occurs in fungal and yeast cell walls, and in the arthropod cuticle in general: the biological composite materials forming the exoskeleton of the lobster *Homarus americanus* and the crab *Cancer pagurus* have shown that all parts of the exoskeleton were optimized to fulfill different functions according to different eco-physiological strains sustained by the animals [37,38,39,40,41,42,43,44,45]. The hard chitinous tissues found in some invertebrate marine organisms are paradigms for robust, lightweight materials. Examples of the superior performances of chitin nanofibrils *in vivo* are the oral grasping spines of Chaetognaths, *Sagitta* in particular, [46,47], and the filaments of the seaweed *Phaeocystis* [48,49]*.* Polyplacophorans, or chitons, are an important group of molluscan invertebrates deemed to have retained many features of the molluscan body plan. They feed on microbes detached from the rocks by the scraping action of the radula, a ribbon-like organ endowed with rows of mineralized teeth. The radular teeth of *Cryptochiton stelleri* are three-fold harder than human enamel [50,51,52,53,54].

In the crystal structures of α- and β-chitins, the chains are organized in sheets and held in place by a number of intra-sheet hydrogen bonds, including the rather strong C–O…NH hydrogen bonds, that maintain the chains at a distance of about 0.47 nm along the a axis of the unit cell. In the α-chitin there are also some inter-sheet hydrogen bonds along the b axis of the unit cell, involving the hydroxymethyl groups of adjacent chains: as already noted, this feature is not found in the structure of β-chitin [55,56,57].

### 1.3. Scope of the Present Review

The scope of this review article is to draw attention to the sudden upsurge of research activity on n-chitin production, development, and biomedical applications. Creative and inspired works dated 2010–2014 are considered here as priority. While n-chitin bearing materials have enhanced mechanical characteristics, it should be underlined that these properties are of outmost importance for biomedical uses of all kinds: for example they permit minimization of the load bearing stress of prosthetic materials, they offer easy handling of surgical aids, and favor cell growth and differentiation.

## 2. Advanced Approaches to the Preparation of Chitin Nanofibrils

### 2.1. Nanochitin Isolation under Mild Oxidative Conditions

In the frame of an early project intended for the preparation of hyaluronan surrogates, chitins were regiospecifically oxidized at C-6 with NaOCl in the presence of the stable nitroxyl radical 2,2,6,6-tetramethyl-1-piperidinyloxy (Tempo^®^, Aldrich, Milan, Italy) and NaBr at 25 °C in water. The obtained anionic oxychitins are fully soluble (pH 3–12); they exert metal chelation, polyelectrolyte complexation with biopolymers such as chitosan, and generate microspheres and beads; they precipitate a variety of proteins, including papain, lysozyme and other hydrolases. Remarkably, 6-oxychitin was assayed for the regeneration of bone [58].

Tempo^®^-oxidized chitin nanocrystals were used to cast films that were fully characterized. They were labeled with a fluorescent imidazoisoquinolinone dye, and simultaneously conjugated with carbohydrate ligands, resulting in dually functionalized nanocrystals. The biorecognition properties of the nanocrystals were probed with lectins and bacteria, resulting in selective interactions with their corresponding cognate carbohydrate-binding proteins. These works represent a new approach to multifunctional nanomaterials based on naturally occurring polymers [59,60,61].

A significant factor that affects transparency of the dispersions, weight ratio of water-insoluble fractions, shape, length, and width of the chitin nanocrystals obtained, is the carboxylate content in the Tempo^®^ oxidized chitins or the amount of NaClO added in the oxidation of α-chitin. The chitin nanocrystals typically had fiber widths smaller than 15 nm, and average widths of 8 nm. The carboxylate groups generated by Tempo^®^-mediated oxidation solely at C6 of the α-chitin at the fibril surface, promote the individualization of the fibrils by mild mechanical treatment. In fact, the preparation of chitin nanofibrils from squid β-chitin by Tempo^®^-mediated oxidation, only requires mild mechanical agitation in water at pH 3–4.8 [62,63].

This was a real breakthrough: since then, the preparation of chitin nanofibrils has been definitely simplified in terms of expenditures, duration, yield, eco-compatibility, and laboratory safety. For instance, the hydrochloric acid concentration was reduced from 3 M to 5 mM; the temperature from 102 °C down to room temperature. Most importantly, in recent works acetic acid has replaced HCl.

### 2.2. Nanochitin Disassembly and Fibrillation by Mechanical and Hydraulic Means

Mechanical treatment under mild acidic conditions is the key to fibrillating dry chitin. The method by Dutta *et al.* includes a chitin powder dispersion in water at 1% followed by addition of acetic acid to adjust the pH to 3 [64]. The slurry is then passed through a high pressure water-jet system equipped with a ball-collision chamber for mechanical disintegration and nano-fibrillation. The resulting n-chitin, further dispersed in water (0.1%), yields films with uniform surface and thickness of *ca.* 25 μm when dried at 40 °C.

Grinders and high speed blenders are also useful for the mechanical conversion of chitin to nanofibers. At pH 3–4, the cationization of amino groups on the fiber surface assists nanofibrillation by exerting an electrostatic repulsive force, in the same way as the carboxyl group generated by Tempo^®^. Even though the degree of deacetylation might be as low as 0.04, the protonation of the amino groups generates repulsion forces that overcome the hydrogen bonds between the nanofibers. The fibrillated chitin samples exhibited uniform structure endowed with high aspect ratio, the fibril width being 10–20 nm. By a grinding treatment, chitin nanofibers were isolated from never-dried crustacean shells after removing inorganics and proteins: equivalent results were obtained. Furthermore, it was confirmed that nanofibers could be conveniently extracted from the natural chitin + protein + inorganic composites of crab shells: on the other hand the acetyl group was not hydrolyzed and the crystalline structure survived intact [65].

α-Chitin powder (<100 μm, acetylation 0.90) was treated to reach a degree of acetylation of 0.70–0.74 by using 33% NaOH in the presence of NaBH_4_ at 90 °C for 2–4 h, with a yield of 85%–90% [66]. The crystallinity index and the crystal size of the α-chitin did not change, showing that deacetylation took place on the crystallite surface. (When 50% NaOH at 90 °C is used, deacetylation occurs instead within the whole mass of the crystallites, thus leading to a decrease of the crystallinity index). Therefore, the said conditions are mandatory for limited and regioselective deacetylation of the crystallite surfaces because, of course, the said treatment with 33% NaOH avoids the generation of highly deacetylated chitosans that disappointingly would dissolve as soon as they are immersed into acidic aqueous media.

The convenient features of the newly developed α-chitin nanofibrils are: (1) commercially available α-chitins can be used as starting materials; (2) nanofibrils are obtained in high yields (85%–90%); (3) the rod-like morphology of the nanofibrils supports the high yields; (4) the α-chitin nanofibril dispersions has high UV-vis transmittance hence high transparency, indicating individualization of α-chitin fibrils; (5) lower costs are involved: no disposal of the brown HCl solution, no handling of enormous quantities of acidic wash water, no cumbersome filtration; (6) the procedure is safer compared with the traditional one and simpler than with Tempo^®^, hence, considering the higher yields, potential applications can be expanded to numerous fields; (7) β-chitins are transformed into α-chitins during treatment with aqueous NaOH, as justified on thermodynamic grounds [67].

### 2.3. Emerging Novel Approaches

Recent investigations still based on the previous hydrolytic approach claimed the isolation of long α-chitin nanofibrils from *Homarus americanus* carapace, suitable for the preparation of nanostructured membranes [68]. Close inspection of the reported data, however, points out the omission of key data from the preparation protocol, such as temperature values and production yields, accompanied by debatable choices such as deproteination protracted for 14 days but leading in any case to partial removal of proteins, depigmentation with the aid of 96% ethanol (instead of more appropriately ethylacetate). Of course unrealistic research planning does not permit economical feasibility studies. Instead, a more pragmatic approach is that followed by Wijesena *et al.* along the lines set forth by the Ifuku group and other researchers [69]: in practice duly cleaned and powdered crab (*Portunus pelagicus)* shells were treated with 2.5 M HCl for 2 days at room temperature under stirring to remove CaCO_3_. After washing, the sample was treated with 1 M NaOH for 2 days at room temperature to remove proteins completely. The pigments were bleached with 3% H_2_O_2_ at pH 10.5 ± 0.5 (Na acetate) for 20 min at 80 °C. The sample dispersed in water (1%, pH 3–4 upon subsequent addition of acetic acid) was sonicated for 2 h to yield a stable bluish dispersion of n-chitin.

The work just mentioned, as well as some others, resulted in the preparation of n-chitosan, besides n-chitin: in fact, Watthanaphanit *et al.* have prepared n-chitosan via deacetylation with NaOH and borohydride, with consequent lowering of the molecular weight to 59 kDa, and the degree of deacetylation to 0.50 [70]; the 1.13% suspension was of colloidal nature. Under milder conditions, Fan *et al.* carrie out the deacetylation of a finely milled chitin powder [66], that yielded viscous birefringent dispersions of n-chitosan at pH values 3–4 [66,71]. Chitin nanofibrils were instead easily produced via gelation of a commercial chitin powder by soaking it at room temperature in the ionic liquid 1-allyl-3-methylimidazoliumbromide, followed by heating at 100 °C. Subsequent sonication gave a chitin dispersion from which chitin nanofibrils were regenerated [72]. When PVA was added, the SEM images of the composite (weight ratio of chitin to PVA 1.0:0.3) provided evidence that the nanofibrillar morphology was preserved. This technique for the preparation of chitin nanofibrils has great advantages compared to the early methods because special equipment and chemical modifications are not necessary.

### 2.4. Self-Assembled Nanofibrils

**Figure 2:**
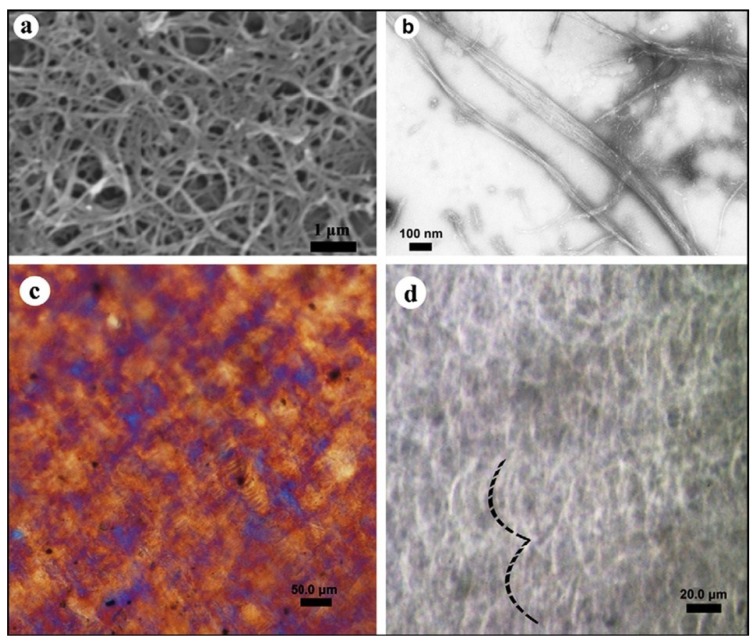
SEM (**a**); TEM (**b**) micrographs of chitosan nanofibrils; and polarized optical microscopy micrographs of chitosan liquid crystal film (**c**,**d**). Reprinted with permission. Copyright © 2011 Elsevier [73].

Despite the desirable nano-dimensional attributes of chitin nanofibrils, abundant surface polar groups cause them to self-assemble upon drying, losing their unique individual nano-domain features. Wet-grinding and high-pressure homogenization were combined to fibrillate chitosan (Φ 50 nm, length *ca.* 1 micron): the obtained nanofiber assembled into a high strength liquid crystal film by self-organization. The thus fabricated transparent liquid crystal film had high tensile strength (*ca.* 100 MPa) and Young’s modulus (*ca.* 2.2 GPa) owing to its ordered, layered structure. The chitosan nanofibrous film presented the characteristic cholesteric texture of a liquid crystal (Figure 2) after self-organization from colloidal suspension [73].

From homogeneous LiCl + dimethylacetamide solutions, chitin nanofibrils precipitated upon the addition of water (10–25 times the original volume). Nanofibers prepared in this fashion generally had a larger diameter (Φ 10.2 ± 2.9 nm) than those prepared from HFIP. However, both types of nanofibers had similar lengths. The resulting nanofibers were made of highly crystalline α-chitin [74]. The crystallization of α-chitin was favored with respect to β-chitin because of the higher number of hydrogen bonds and higher thermodynamic stability of α-chitin [67].

**Figure 3:**
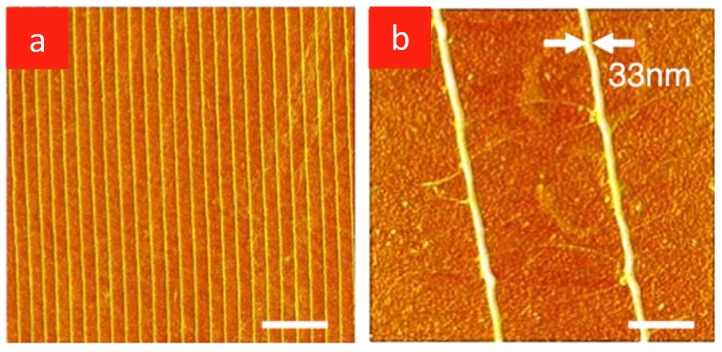
Microcontact printing of chitin nanofibers onto glass supports. Topographic AFM images of chitin nanofiber patterns printed from 0.05% (w/v) ink. The PDMS stamp is replicated from a holographic diffraction grating with 1200 grooves/mm. Scale bars: (**a**) 4 μm and (**b**) 400 nm. Reprinted with permission. Copyright © 2011 Elsevier [76].

To mimic the association of chitin with proteins *in vivo*, silk was added to solutions of squid pen β-chitin in HFIP, which were dried to yield homogeneous films made of ultrafine (~3 nm) chitin nanofibrils embedded in a silk fibroin matrix [75]. The chitin nanofiber content of the biocomposite was easily tunable by varying the chitin/silk ratio in solution. This desirable feature afforded a simple strategy to fine-tune the biocomposite properties. Transparent chitin-silk films co-assembled from solution were easily manipulated with soft-lithography skill to manufacture optical devices such as diffraction gratings (Figure 3) [76,77].

**Figure 4:**
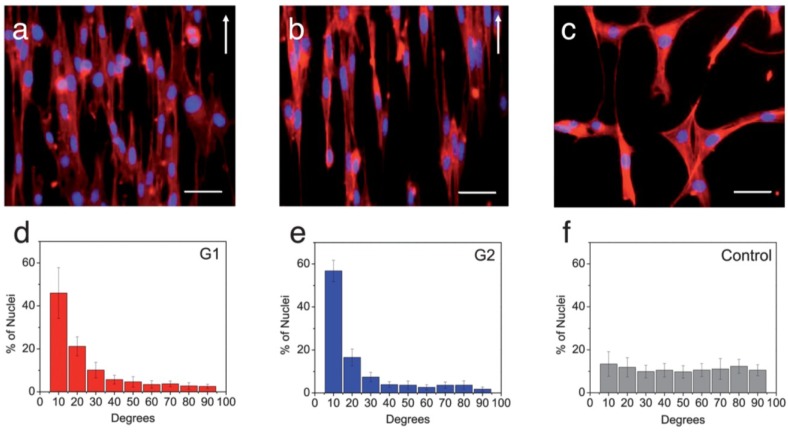
Fluorescence images of the actin cytoskeleton of the cells on: (**a**) G1; (**b**) G2 support; and (**c**) control sample after five days of culture. Scale bars 50 μm. The white arrow shows the longitudinal direction of the patterns; (**d**–**f**) Distribution of alignment angles of cell nuclei on the patterned samples and control. Note: G1 is a pattern with 3.2 μm spacing (smaller than the average cell diameter) and G2 with 11.2 μm spacing (slightly larger than the average cell diameter). Reprinted with permission. Copyright © 2013 Elsevier [78].

On the chitin micropatterned supports, cells with a spindle-like morphology aligned their cytoskeleton along the major axis of the pattern features (contact guidance, Figure 4a,b). In contrast, the cells grown on the control chitin supports had no preferred orientation (Figure 4c). After 5 days of cell culture a larger proportion of the cells aligned within the 0–10° preferred angle (Figure 4d,e) as opposed to control (Figure 4f). This proof-of-concept data showed the potential of self-assembled chitin nanofibers to create robust and flexible micropatterned materials intended for tissue engineering, that in any case need to be easily retrievable, to be mechanically robust to provide substantial support for the generation of new tissues, and to maintain proper handling characteristics [78]. The versatility of chitosan-based nanofibers with controllable size and directional alignment as well as highly ordered and customized patterns was also demonstrated by Fuh *et al.* who used PEO-chitosan nanofibers [79]. Analogous investigations were carried out on the manufacture of chitin nanofiber templates for artificial neural networks: chitin nanofiber surfaces were deacetylated to form chitosan nanofibers (Φ 4 nm and 12 nm) that were coupled with poly-d-lysine. In fact, the 4 nm chitosan nanofibers with poly-d-lysine supported 37.9% neuron viability compared to only 13.5% on traditional poly-d-lysine surfaces after 7-day culture [80].

A facile freeze-drying approach to assemble chitin nanofibers (Φ 20 nm) into a variety of structures whose size and morphology are tunable by adjusting freezing temperature and heat transfer characteristics was adopted by Wu J *et al.* [81]. Notwithstanding the fact that the described self-assembly has proven to be viable, it would be better to use solvents exempt from toxicity and volatility. One approach is based on ionic liquids considered as green solvents capable to replace organic compounds [82], whilst the second approach is based on urea in alkaline solution [83,84,85,86]. In addition, self-assembled chitin nanofibers are important for the study of randomly oriented nanofiber networks. 

### 2.5. Chitin Foams and Transparent Films Obtained by Sonication

A transparent nanofibril suspension is readily obtained by sonicating purified squid pen powder in water. The β-chitin nanofibrils were 3–10 nm in width and several microns in length, with acetylation degree of 0.84, *i.e.*, 10% lower than controls. The suspension could be transformed into stable 3-D hydrogel by simply heating at 180 °C for 1–4 h in an autoclave. Experimental evidence was provided that hydrophobic interaction between fibrils has a role in self-assembling into hydrogels. The physical properties of the hydrogels could be easily modulated. The suspension of 1% (w/v) fibrils (4 h, 180 °C) generated a hydrogel with 99.3% water content, fibrils with 87% crystallinity and mechanical strength of 0.7 N breaking force. The chitin hydrogels, molded into desired shapes and prepared without chemical crosslinkers, could be of interest for wound dressing and tissue engineering [87].

**Figure 5:**
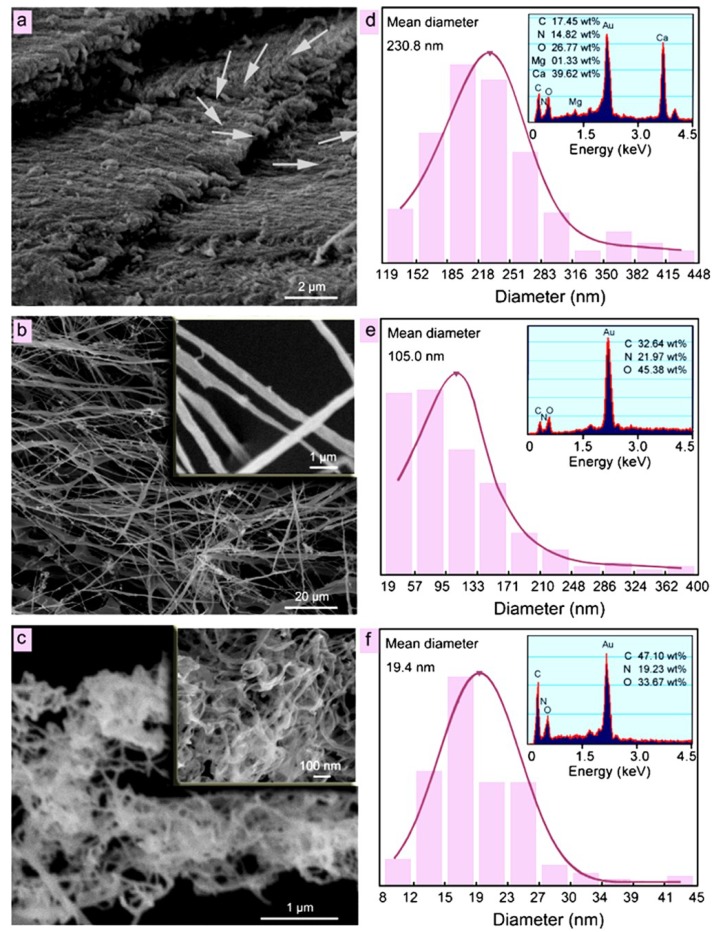
SEM micrographs of (**a**) dry shell; (**b**) after 5 min sonication; and (**c**) after 30 min sonication; (**d**–**f**) are the distributions of the fiber diameters respectively, and insets are EDS spectra. Reprinted with permission. Copyright © 2013 Elsevier [94].

Likewise, α-chitin nanofibers were fabricated with dried shrimp shells via a simple high-intensity ultrasonic treatment (60 KHz, 300 W, pH = 7). The diameter of the obtained chitin nanofibers could be controlled within 20–200 nm by simply adjusting the sonication time. This high-intensity sonication treatment separates nanofibers from natural matrices after the purified chitin is dispersed in water. The pulsed sonication disassembled chitin into high-aspect-ratio nanofibrils with a uniform width (19.4 nm after 30 min) (Figure 5). The α-chitin crystalline structure and molecular structure were preserved; interestingly, ultrasonication slightly increased the degree of crystallinity. Furthermore, highly transparent chitin films (transmittance 90.2% at 600 nm) and flexible ultralight chitin foams were prepared from chitin nanofiber hydrogels.

Optically transparent nanocomposites using chitin nanofibers with acrylic resin were prepared to examine the fibrillation process of dry chitin, and the nanofiber homogeneity. Since dried chitin was fully fibrillated, the optical losses of these composites were less than 2% even after the drying process [88,89]. The nanofibrils were small enough to preserve the transparency of the neat polymethylmethacrylate resin; furthermore the extremely low thermal expansion of nano-chitin films improved the thermal stability of PMMA [90,91] even in the presence of glycerol [92]. Nano-chitin transparent films are thermally stable up to 200 °C for a long period of time [93].

Transparent and highly viscous liquids were obtained by sonication of the partially deacetylated chitins in water at pH 3–4. Transmission electron microscopy revealed that the liquids consisted mainly of independent nanofibrils with average width and length 6–7 nm and 100–200 nm, respectively. In fact, some α-chitin nano-fibrils of >500 nm in length were present. Because the acetylation values 0.70–0.74 correspond to 1.34–1.56 mmol/g amino group content, positive charges form on the α-chitin fibril surfaces in high density by protonation in water at pH 3–4 [66].

The ultrasonication used for physical fibrillation, could disassemble the micro-fibres into nanofibrils in water via cavitation. The shockwaves, generated by the implosion of the cavity, break the relatively weak chitin interfibrillar hydrogen bonding and the Van der Waals forces to gradually yield nanofibrils [94]. The method of degradation of chitosan by hydrodynamic cavitation, has some advantages such as a high degradation rate, low energy consumption, equipment which is easy to obtain, the ability to handle large quantities of sample, and no pollution risk to the environment [95].

## 3. Manufacture of Chitin Nanofibers via Electrospinning

Recent reviews on spinning methods and e-spun fibers testify the increasing interest in this technology, notwithstanding the high costs of the solvents and the challenges faced in scaling-up [96,97,98,99,100]. It should be underlined that the term nanofibers refers to fibers with diameters in the nano range and undefined length, whilst the terms nanofibrils, nanocrystals and whiskers refer to highly crystalline objects having both diameter and length in the nano range, unless otherwise specified.

### 3.1. Electrospinning of Chitin

Besides the already mentioned *N*,*N*-dimethylacetamide + LiCl, other solvents such as hexafluoroacetone, hexafluoro-2-propanol, methanol and ethanol saturated with calcium chloride dissolve chitin which can then undergo e-spinning when accompanied by some other polymer; moreover, chitin nanofibrils can be incorporated into certain e-spinnable compounds.

For example, in the work of Junkasem *et al.* fiber mats of e-spun poly(vinyl alcohol) (PVA) were made containing α-chitin nanofibrils prepared from shrimp shells. The as-prepared chitin nanofibrils exhibited lengths in the range 231–969 nm and widths in the range 12–65 nm. The incorporation of chitin nanofibrils within the as-spun fiber mats increased the Young’s modulus by 4–8 times over that of the neat as-spun PVA fiber mat. The maximum tensile strength 5.7 ± 0.6 MPa was obtained when the chitin to PVA ratio was ca. 5.1%. Similarly, composite nanofibers based on PVA and nanocrystals of α-chitin (Φ *ca.* 31 nm, length *ca.* 549 nm) have been prepared (Φ 175–218 nm). The addition itself and the increase of the amount of the nanofibrils caused the crystallinity of PVA within the nanocomposite materials to decrease and the glass transition to increase [101,102]. We can best appreciate these results if we recall that certain early patents on chitin surgical sutures obtained by wet spinning chitin in dimethylacetamide + LiCl could not be exploited solely because the fibers scarcely had knot resistance [103,104].

To manufacture biodegradable scaffolds, chitin + poly(glycolic acid) nanofibers e-spun in hexafluoro-2-propanol (Φ *ca.* 140 nm) were investigated: the nanofibrous mats with PGA to chitin ratio 1:3 (and with bovine serum albumin coating) were the best in terms of cell attachment and spreading for normal human fibroblasts [105]. *In vitro*, the blend fibers degraded faster than pure PGA fibers at pH 7.2. Shalumon *et al.* developed e-spun carboxymethyl chitin + PVA blends that were exposed to glutaraldehyde vapors to make them water insoluble, *i.e.*, suitable for tissue engineering applications [106].

It is important to underline that the surface area of n-chitin e-spun mats is definitely smaller than the surface area of n-chitin aerogels obtained from hydrogels submitted to low-power ultrasonication, and dried by using supercritical CO_2_ (surface area 58–261 m^2^/g). Chitin was also fabricated into nanoporous aerogels by using aqueous NaOH + urea chaotropic solution as solvent (surface area up to 366 m^2^/g) [107,108].

### 3.2. Electrospinning of Chitosan

Review articles dealing with this specific topic are available [109,110,111,112]. Nanofibrous mats were produced by e-spinning of chitosan + poly(ethylene oxide) (PEO) solution. In the presence of poly(hexamethylene biguanide), thinner fibers (Φ 60–240 nm) were obtained [113]. Chitosan and alginate were e-spun together with the aid of a dual jet system: PEO was applied to increase the viscosity of polymer solutions, and to yield nanofibers with appropriate morphology [114].

Novel biodegradable sutures were manufactured via coaxial e-spinning. Poly(lactic acid), a hydrophobic polyester, has good biocompatibility and mechanical properties, therefore, e-spun poly(lactic acid) nanofibers with uniaxial alignment were coated with chitosan and applied as tissue sutures: *in vivo* they exhibited better histocompatibility than silk, optimum mechanical resistance in clinical trials, and promoted cell growth. It is important to underline that they exhibited tensile and knot strengths similar to those of a commercial suture [115]. Some works have been devoted to the manufacture of PLA + chitosan composites, among which the one by Li YJ *et al.* who used Na lauryl sulfate to promote the distribution of chitosan in the periphery of the nanofibers; these materials supported growth and attachment of mouse fibroblasts [116].

It should also be considered that the preparation of nanostructured scaffolds is based on the chemical and morphological transformation of chitin nanofibrils into highly deacetylated n-chitosan. Upon deacetylation the chitin nanofibrils yield a colloidal chitosan solution, whose viscosity increase suggests that the n-chitosan network behaves as a polymer bulk in the solution. Facile chemical modifications of the chitosan nanoscaffold with lactose or maltose leads to white fluffy mesoporous chitosans whose characteristic properties (surface area, pore size, zeta potential) are quite suitable for tissue engineering [117].

**Figure 6:**
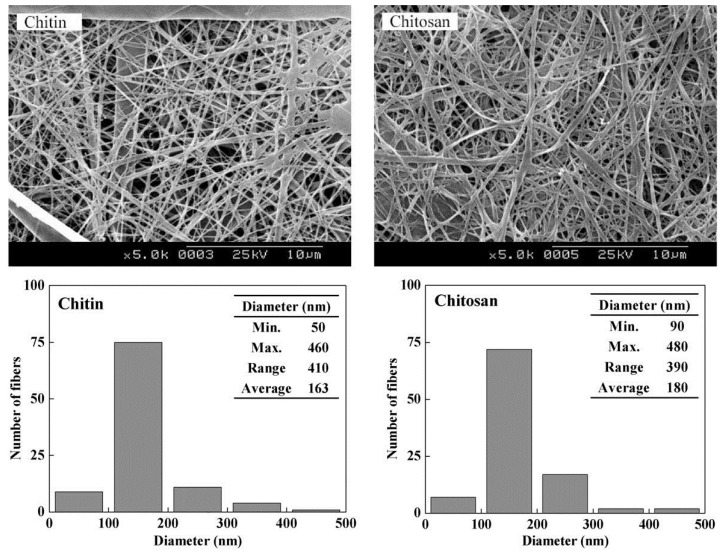
Electrospun mats of nano-chitin (left) are transformed into mats of nano-chitosan (right) by chemical deacetylation up to 0.85, without significant dimensional change (no shrinkage). Reprinted with permission. Copyright © 2004 Elsevier [118].

It is also true that the preformed nanofibrillar mats of n-chitin can be deacetylated: in this case n-chitin becomes n-chitosan, but the texture remains practically the same as shown in Figure 6 [118], and amply confirmed by Pereira *et al.* [119].

Further utilization of chitosan nanofibrous mats e-spun from chitosan solutions in trifluoroacetic acid with or without chloromethane as the modifying co-solvent was found to be limited by the loss of the fibrous structure when the mats were in contact with neutral or weak basic aqueous solutions due to complete dissolution of the mats [120]; the effect of the degree of deacetylation was also studied [121]. Much improvement in the neutralization method was achieved with a saturated Na_2_CO_3_ solution. Torres-Giner *et al.* also developed porous e-spun chitosan nanofibers by using chloromethane [122].

The second solvent system demonstrated to effectively produce nanofibers was 30% acetic acid: thin nanofibers (Φ 40 nm) were produced together with large beads, but at 90% concentration the fiber’s diameter increased to 130 nm without bead formation. Thus uniform mats of 130 nm fibers were obtained by e-spinning aqueous 90% acetic acid containing 7% chitosan in a 4 kV/cm electric field. These studies showed that the acetic acid concentration in water strongly influenced the surface tension of chitosan solutions [123]. Net charge density of the chitosan solution linearly increased with acetic acid concentration in water resulting in more charged ions available for charge repulsion. A fibrous scaffold comprising chitosan and PCL was e-spun from mixture of formic acid and acetone [124]. The PCL e-spun mats exhibited hydrophilicity when the n-chitin content was >25 wt% [125].

Rosic *et al.* demonstrated that interfacial, rather than bulk, rheological parameters can be used to predict the results of the e-spinning process [99]. By using the interfacial parameters (viscosity, G′, G″) of samples with homologous compositions, solutions of chitosan and PEO with differences in polymer ratio that form smooth nanofibers, can be identified. The bulk parameters are determined by polymer concentration and directly affect jet initiation, while the interfacial parameters determine the continuation of the jet and fiber formation.

Microfluidic-based pure chitosan microfibers (*ca*. 1 m long, Φ 70–150 μm) were fabricated without any chemical additive, and hepatoma HepG2 cells were seeded onto them. The functionality of the hepatic cells cultured on chitosan microfibers, analyzed by measuring albumin secretion and urea synthesis, was satisfactory; the microfiber chip could be useful for liver regeneration [126,127].

### 3.3. Electrospinning of DBC-Chitin

Dibutyryl chitin (DBC), a lipophilic chitin diester, is fully soluble in various organic solvents, thus it has been processed into fibers via conventional dry spinning from acetone, and wet spinning from dimethylformamide. Fine DBC fibers (Φ 300 nm) have been e-spun from ethanol and then regenerated with alkali for the preparation of nonwoven wound dressings. Although the preparation of DBC was initially made with the aid of perchloric acid, the adoption of less dangerous acids has simplified the preparation and made possible the scaling-up of the production. The most favorable characteristics of DBC are: hemocompatibility, lack of thrombogenicity, full biocompatibility toward human fibroblasts and keratinocytes, as well as documented technical feasibility of medication items such as plasters and dressings [128,129,130,131,132]. The effect of e-spun non-woven mats of dibutyryl chitin + poly(lactic acid) blends and other formulations were assayed for the purpose of wound healing [133,134,135].

## 4. Biomedical Applications of Nano-Chitins

Major biomedical applications of chitin and chitosan nanofibers have been recognized as emerging in the areas of tissue engineering, wound dressing, cosmetic and skin health, stem cell technology, anti-cancer treatments, drug delivery, anti-inflammatory aids, antimicrobial agents, biosensors, and reduction of obesity [136,137]. Nano-sized materials of interest for pharmaceutical uses have been reviewed as well as polysaccharide nanocarriers for therapeutic applications [138,139]. The state of the art and perspectives of the hemostatic polymers have been reviewed [140].

### 4.1. Hemostasis and Wound Healing

Chitin nanofibrils Talymed^®^ manufactured from marine diatoms by Marine Polymer Tech., MA, USA, have an average length of *ca.* 80 μm. Gamma irradiation resulted in length shortening down to 4–7 μm, width of 100–150 nm, and thickness of 40–60 nm. Treatment of cutaneous wounds with the said FDA approved n-chitin (mainly used for hemostasis) resulted in more rapid wound closure in diabetic animal models, owing to increased expression of several cytokines, growth factors, and innate immune activation, besides activating angiogenesis, cell proliferation and re-epithelialization, as known long since [141].

Standard care for venous leg ulcers has remained unchanged over several decades despite high rates of initial treatment failure and ulcer recurrence. Kelechi *et al.* evaluated the efficacy, safety, and tolerability of Talymed^®^ among patients with leg ulcers [142]. In a randomized, multi-center study, 86.4% of patients experienced complete wound healing when receiving multilayer compression plus Talymed^®^ *vs.* 45% receiving multilayer compression alone. The pilot study suggested that n-chitin is well tolerated and effective. Results reported by Fischer *et al.* provide evidence that n-chitin nanofibers activate platelets and accelerate the clotting of blood, and on how best to achieve surface hemostasis when patients are coagulopathic because of shock or treatment with antiplatelet drugs [143].

n-Chitin promoted the expression of α- and β-defensins in endothelial cells and β-defensins in keratinocytes. Lindner *et al.* assessed the antimicrobial efficacy of n-chitin [144]: *S. aureus* infected wounds were either treated with n-chitin or left untreated for three and five days. Animals treated with n-chitin showed significant reduction in Gram positive staining by day 5 post wounding as compared with untreated wounds. Given that defensins are part of the innate immune system, activation of these pathways precludes the generation of resistant organisms and also allows for the antibiotic-independent clearance of bacterial infection. Thus they concluded that n-chitin enhances wound healing while concomitantly limiting wound infection. Antimicrobial n-carriers such as n-emulsions, n-liposomes, n-particles and n-fibers were reviewed by Blanco-Padilla *et al.* [145].

The reasons for the exceptional performances reside in the chemical nature and tertiary/quaternary structure of the chitin/chitosan whose surface is recognized by the platelets: via the staining of the activation marker P-selectin, it was possible to reveal the sudden activation of the platelets whose pseudopodia make a robust contact immediately upon activation [146]. The influence of altered platelet function by treatment with the ADP inhibitor Clopidogrel^®^ (Sanofi, Paris, France) on wound healing and the ability of n-chitin to repair wounds in diabetic mice treated with Clopidogrel^®^ were studied by Scherer *et al.* [147]. With the growing number of patients being treated with antiplatelet drugs, chitin nanofiber mats may be useful more generally for enhancing wound healing and restoring homeostasis.

Vacuum-assisted closure has become the preferred mode to treat many complex wounds, but could be further improved by methods that minimize bleeding and facilitate wound epithelialization. Membranes consisting entirely of chitin nanofibers were applied to dorsal excisional wounds of db/db mice followed by application of a vacuum device. Results included significant expression of PDGF, TGF-β, EGF, superior granulation tissue formation rich in collagen-I as well as epithelialization and wound contraction. Thus the application of membranes containing n-chitin, prior to the application of the polyurethane foam interface of vacuum devices, leads to most satisfactory healing and represents an advantage that reduces the risk of bleeding [148].

### 4.2. Joints and Bones

The early stages of degenerative disc disease primarily affect the disc nucleus pulposus. The product used by Gorapalli *et al.* was a relatively high MW n-chitosan [149]. The nanofibers were then manufactured as thin membranes by simple filtration and drying. Membranes were suspended in 40% NaOH at 80 °C for 3 h for deacetylation. Gel 1 consisted of 33.3 mg/mL of a sulphated n-chitosan having approximately 60%–70% of the sugar residues sulphated and 70% deacetylated (~500 kDa), plus 30 mg/mL of n-chitosan (pH 8.3). Gel 2 consisted of 10 mg/mL of irradiated (LMW) n-chitin and 30 mg/mL of n-chitosan (pH 6.8). These shortened nanofibers were 5–7 μm in length instead of 80 μm of the original nanofibers. Gel formulations were developed so as to be injectable into the nucleus pulposus of the intervertebral disc. The sulphated, deacetylated n-chitin hydrogel demonstrated most evident biological effects on cell viability, metabolic activity, and proteoglycan expression. RT-PCR and immunohistochemical data corroborated the expression of characteristic disc markers, aggrecan and collagen type II in cultured cells. The sulphated n-chitosan hydrogel possesses convincing characteristics, although results cannot yet be extrapolated to *in vivo* situations [149].

Muise-Helmericks *et al.* drilled circular holes (Φ 2.0 mm) in the femurs of rabbits and implanted n-chitin [150]. All the 15 n-chitin test sites were found to have new bone tissue present. Hematoxylin and eosin histology of the n-chitin-treated sites showed the presence of osteoblasts, osteocytes, and trabeculae of new bone at the end of the 28-day study period. The n-chitin mats activated the regeneration of new bone tissue in a rabbit model after 28 days of implantation, whereas the control bone did not. It is known that large bone defects do not heal unless treated with suitably modified chitosans, the plain chitosan itself being inactive [151,152,153]. A totally original approach was adopted in a bio-inspired work by Malho *et al.* who used a genetically modified protein to functionalize superficially the chitin nanofibers with a mineralization domain [154]. The said protein has two distinct groups, the first one (for binding chitin) the chitin-binding domain of bacterial chitinases, and the second one (for binding minerals) a fragment of aspein, a shell-specific protein from the oyster *Pinctada fucata* [155]. The charge density enabled the combination of CaCO_3_ crystals with the chitin, thus providing a composite material endowed with enhanced mechanical and biochemical properties, probably suitable for bone surgery.

### 4.3. Ulcers and Traumatic Wounds

Azuma *et al.* found that n-chitin alleviates the clinical symptoms and suppresses the onset of ulcerative colitis in an animal model [156], in agreement with Ifuku *et al.* [10]; furthermore, n-chitin suppressed myeloperoxidase activation in the colon and decreased serum interleukin-6 concentration. In contrast, the application of chitin powder did not produce any anti-inflammatory action. Actually, 5-aminosalicylate is the only drug available, not exempt from side-effects [157,158,159]. Further work showed that α-chitin nanofibrils decreased positive areas of NF-kB staining in the colon tissue (7.2 in the chitin nanofibrils group *vs.* 10.7%/fields in the control). Chitin nanofibrils also decreased serum monocyte chemotactic protein-1 concentration in ulcerative colitis (24.1 in the chitin nanofibrils group *vs.* 53.5 pg/mL in the control). Moreover, the nanofibrils depressed the positive areas of Masson’s trichrome staining in colon tissue (6.8 in the chitin nanofibrils group *vs.* 10.1%/fields in the control) [160].

Chitin nanofibrils suspended in water were incorporated into chitosan glycolate upon simple addition of the proper amount of chitosan powder and glycolic acid crystals. No aggregation or precipitation of chitin nanofibrils was observed in the course of several months. The wound dressing included a flexible support made of non-woven dibutyryl chitin (0.8 g), chitosan glycolate solution 1.0% (8 g), containing chitin nanofibrils (2 g/L), and chlorhexidine at physiological pH. The preparations were freeze-dried and sterilized. Ulcers and wounds in 75 patients were treated for 60 days. In the treatment of torpid and slowly healing lesions or ulcers, the association of the chitin + chitosan gel with a foam-like dressing induced physiological repair. A remarkable advantage was that the glycolic acid solutions are capable to preserve chitosan from microbial spoilage. Moreover, the nanofibrillar chitin + chitosan glycolate composites exert control over certain biochemical and physiological processes, besides hemostasis: while chitosan provides moderate antimicrobial activity, cell stimulation and filmogenicity, chitin nanofibrils slowly release *N*-acetylglucosamine, and recognize growth factors [161]. In fact, the introduction of chitin nanofibrils (0.1%–0.3%) in a chitosan solution influences the orientation of the chitosan macromolecules and leads to an increase in the strength and Young modulus of the chitosan + n-chitin composite fibers. The orientation of the chitosan macromolecules on the surface of the chitin nanofibrils was confirmed by the molecular dynamics simulation of systems containing one chitosan molecule on the chitin nanocrystallite surface [24,162].

Rod-like chitin nanofibrils were used to reinforce chitosan films, and composite membranes were prepared by casting/evaporation. The films with 3% chitin content had a tensile strength 2.8 times higher than that of plain chitosan film. Sterilized transparent film disks (Φ 7 cm) were placed on the inoculated media: after 24 h at 37 °C the composite film effectively inhibited all of the three strains tested, namely *S. aureus*, *E. coli* and *Corinebacterium michiganense*, thus providing preliminary indications on its suitability as an antibacterial film [163,164]. Genipin, as a crosslinker of the chitosan film assures the endurance of the composite in the culture environment for a prolonged period of time [165]. Chitosan films reinforced with chitin nanofibrils in admixture with tannic acid were conveniently crosslinked via amide formation by simply keeping them at 105 °C for 90 min [166].

Therefore, the presence of chitin nanocrystals into chitin fibers (Φ 223–966 nm) had a positive impact on the mechanical properties of e-spun mats further crosslinked with genipin. Those with a tensile strength of 64.9 MPa, modulus of 10.2 GPa and surface area 35 m^2^/g were considered for wound dressing. The water vapor transmission rate of the mats was between 1290 and 1548 g m^−2^day^−1^, and was in the range suitable for injured skin or wounds. The e-spun fiber mats showed compatibility with adipose derived stem cells [167].

According to Tchemtchoua *et al.* the best concentrations for e-spinning were 7.9% medical-grade chitosan and 1% PEG (900 kDa) in 6.5% acetic acid. The chitosan nanofiber mats produced by e-spinning were compared with evaporated films and xerogels. The nanofibrillar structure strongly improved cell adhesion and proliferation *in vitro*. When implanted in mice, the nanofibrillar scaffold was colonized by mesenchymal cells and blood vessels. When used as a dressing, covering full thickness skin wounds in mice, chitosan nanofibres induced a faster regeneration of both epidermis and dermis. They shortened the healing time of skin wounds by stimulating migration, invasion, and proliferation of the relevant cutaneous resident cells [168].

### 4.4. Dermal Protection

Stable PCL + chitin nanofibrous mats were manufactured from blended solutions of PCL and chitin dissolved in the co-solvent hexafluoro-2-propanol and trifluoroacetic acid [169]. The PCL-chitin nanofibrils (Φ 340 nm) showed decreased water contact angles as the proportion of chitin increased. Instead, the tensile properties of mats containing 30%–50% wt/wt chitin were enhanced compared with PCL mats. The viability of human dermal fibroblasts in culture for up to seven days was higher on composite than on PCL nanofibrous mats, with viability directly correlated with chitin concentration. Therefore PCL-chitin nanofibrous mats are useful as implantable substrates to modulate the viability of human dermal fibroblasts [169].

Ito *et al.* suggested that the application of n-chitin to skin, protects the epithelial cells, owing to its capacity to prevent moisture evaporation [170]. Moreover, the n-chitin (pH 6) group scored higher on histological data after the first 8 h of application, indicating that n-chitin is a very effective dermal protector in the long term even in comparison with GlcNAc. Thus n-chitin protects and hydrates the skin while not being allergenic.

## 5. Biomedical Applications of n-Chitosan

From the standpoint of this review article, a major difference between n-chitin and n-chitosan resides in the capacity of the latter to form polyelectrolyte complexes owing to its high cationicity. Polyelectrolyte complexation is an effective strategy for enhancing the mechanical properties of nanofibrous scaffolds. For the improvement of the mechanical and biochemical properties of chitosan intended for pharmaceutical and biomedical applications, blending with other polymers is important, since the native ECM is a complex of polysaccharides and proteins with nanofibrous porous structure. Moreover, crosslinking is often adopted, in particular with the use of genipin, a commercially available plant pigment ten thousand fold less cytotoxic than glutaraldehyde; moreover n-chitosan promptly undergoes other reactions such as reductive amination of aldehydes and ketones [171].

A number of drugs and other significant compounds can be added to the chitosan solutions for incorporation in the nanofibers in the case of e-spinning. For example, *Garcinia mangostana* extracts were incorporated into chitosan + EDTA + PVA solution and e-spun. The fibrous mats exhibited antioxidant and antibacterial activity and accelerated the wound healing process [172]. Polyethylene terephthalate fibrous mats e-spun in the presence of honey were proposed as potential wound dressings owing to their improved characteristics besides their suitable structural properties [173].

### 5.1. Epithelial Tissue Regeneration

Polyelectrolyte complexation enhances the mechanical properties of the nanofibrous scaffolds [174]. With the aid of the e-spun chitosan + gelatin model, it was measured that the storage modulus of a nanofiber mat is at least one thousand-fold higher than that of plain chitosan or gelatin membranes. Further, the annealing process promotes the conjugation of the oppositely charged polymers and thus the tensile modulus becomes 1.9-fold more elevated. When the molar ratio of the anhydroglucosamine units in chitosan to carboxyl units in gelatin was 1:1, the complex nanofiber mats displayed the highest mechanical strength. The characteristic properties of chitosan-gelatin edible films were reported by Jridi *et al.* and Tsai *et al.* [175,176].

Collagen has been widely used for tissue-engineering, in consideration of its biological origin, and ubiquitous presence in living organisms. Chitosan forms polyelectrolyte complexes with collagen: for example, chitosan + collagen composites were prepared by dissolving collagen in HFP, and chitosan in HFP + TFA [177,178]. The samples were e-spun at room temperature, and crosslinked with glutaraldehyde vapor. The authors examined the expression of ICAM-1 and VCAM-1, for which the chitosan + collagen scaffold can act as a stimulus. ICAM-1 and VCAM-1, adhesive proteins of the immunoglobulin superfamily, occur on the endothelial cell membrane and regulate the adhesion process of the leucocytes to these cells: they were used as primers for RT-PCR. Results showed that the chitosan + collagen nanofibrous scaffold enhanced the attachment, spreading, and proliferation of porcine iliac artery endothelial cells. The endothelial cells grown on the e-spun chitosan + collagen scaffolds maintained the capacity to regulate the cell cycle via the p53 mRNA expression and hence to function as tumor suppressors. E-spun chitosan mats coated with collagen were proposed for skin engineering [179] and curcumin-loaded mats were studied for wound healing [180]. Du *et al.* mimicked the natural blood vessel microenvironment by using heparinization and immobilization of vascular endothelial growth factor in the chitosan + PCL. As a consequence, the anti-thrombogenic properties of these scaffolds were enhanced [181].

Catalase, a redox enzyme, is generally recognized as an efficient agent for protecting cells against hydrogen peroxide. The immobilization of catalase was accomplished by depositing the PEC made of cationic chitosan and anionic catalase on e-spun cellulose nanofibrous mats via e-spinning and layer-by-layer deposition. The immobilized catalase protected cells against cytotoxic effects produced by H_2_O_2_ as demonstrated by TEM and SEM [182].

Another protein of interest is fibroin: the silk fibroin + chitosan composite nanofibers (Φ 185.5–484.6 nm) were easily e-spun in mixtures of hexafluoro-2-propanol and trifluoroethanol. Because the fibers as such are promptly water-soluble, they were then crosslinked with glutaraldehyde. The fibroin helps e-spin chitosan and contributes to the biocompatibility. The mats were found to promote the attachment and proliferation of murine fibroblasts [183].

**Figure 7:**
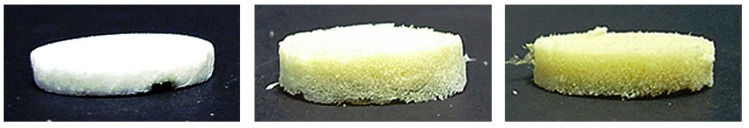
Appearance of (left) n-cellulose, (middle) n-chitin, (right) n-cellulose + n-chitin + sericin. The latter contains glutaraldehyde. Reprinted with permission. Copyright © 2014 Elsevier [184].

Composites of n-cellulose, n-chitin, and sericin were developed by Ang-atikarnkul *et al.* [184]: n-cellulose was used because of its nanofibrillar structure with high aspect ratio. n-Chitin was chosen as addtional component owing to its ability to promote the repair of wounds. The n-cellulose + n-chitin + sericin scaffolds with different compositions were prepared by freeze-drying and then treated with glutaraldehyde vapor (Figure 7). The release of the sericin from the nanocomposites was assessed in terms of blend composition, presence of lysozyme, and NaCl concentration. Sericin, a glue-like protein found in silk cocoons, was found to have good moisturizing and antioxidant capacity.

The water-soluble *N*-carboxyethyl chitosan was synthesized straightforwardly via a Michael addition reaction of chitosan with acrylic acid. Because of unsatisfactory e-spinning of aqueous mixtures of *N*-carboxyethyl chitosan and silk fibroin, PVA was selected as a polymeric additive to produce e-spun nanofibrous mats owing to its good fiber-forming, biocompatibility, and chemical resistance properties. Indirect *in vitro* cytotoxicity assessment of e-spun nanofiber mats with mouse fibroblasts indicated good biocompatibility. These composites might be assayed for wound dressing [185]. Fibroin-chitosan scaffolds with aligned nanofibrous structures were fabricated by Dunne *et al.* to guide vasculature in tissue engineering and repair [186]. The use of nanofibers for cardiovascular tissue engineering was discussed by Oh & Lee [187].

It is also interesting to note that n-chitosan can accompany other polysaccharides. For example, e-spun chitosan nanofibers were deposited onto cellulose treated with atmospheric plasma to generate a composite bandage with higher adhesion, better handling properties, enhanced bioactivity, and suitability for moisture management [188].

Repeated freeze/thaw cycles induced packing and phase separation of the hydrogels made of hemicelluloses, chitin nanofibrils and PVA (average length *ca.* 200 nm, width 40 nm). Large pores led to remarkable swelling, whereas the formation of a compact structure resulted in good mechanical strength and thermal stability. The chitin nanofibrils were embedded homogeneously in the PVA + hemicellulose matrix and limited the packing process, leading to the improvement of compressive strength and crystallinity of the hydrogels [189,190]. Analogous work was done on n-chitin incorporated into carboxymethylcellulose films [191].

Cytocompatible composite films were prepared by adding chitin nanofibrils to a cellulose solution in NaOH + urea. HeLa and T293 cells seeded onto the surface of the said films showed absence of toxicity to both cell types, and cell adhesion and proliferation were enhanced by chitin nanofibrils [192]. Experimental observations were reported in the case of ulvan + chitosan + PEG nanofibrous mats obtained with a strongly acidic polysaccharide from the plant *Ulva rigida*. The nanofibrous structure of these constructs, mimicking the fibrous part of the extracellular matrix, favored the excellent attachment of the osteoblasts [193].

### 5.2. Bone and Dental Tissue Regeneration

**Figure 8:**
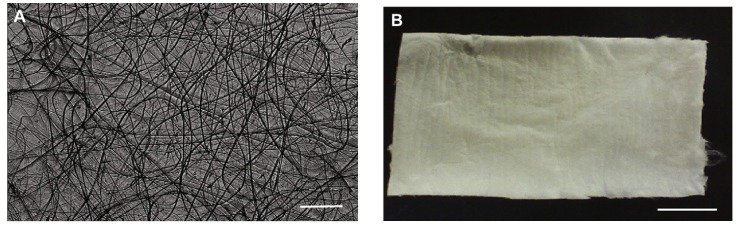
Electrospun chitosan microfibers (**A**); and the fibrous mat (**B**). Scale bar for (**A**) is 200 μm, and for (**B**) is 1 cm. Reprinted with permission. Copyright © 2012 Elsevier [196].

Tan *et al.* demonstrated that chitosan increases collagen-I and osteopontin expression, promotes osteoblast proliferation and osteogenesis in mesenchymal stem cells, and reduces osteoclastogenesis [194]. Norowski *et al.* prepared e-spun chitosan mats crosslinked with minor amounts of genipin, and showed that the obtained mats were cytocompatible and supported fibroblast cell proliferation for nine days; they did not activate monocytes to produce nitric oxide *in vitro* in the absence of lipopolysaccharide [195]. As a consequence of high surface area, decreased crystallinity degree (−14%), and decreased molecular weight of chitosan (−75%), the ultimate suture pullout strength was significantly lower (−51%) than that of commercially available collagen membranes. However, when genipin was used to crosslink nanofibrous chitosan made to incorporate hydroxyapatite nanoparticles, the Young’s modulus value of the composite was 142 ± 13 MPa, which is similar to that of the natural periosteum. Pure chitosan and hydroxyapatite-chitosan composites supported adhesion, proliferation and osteogenic differentiation of murine 7F2 osteoblast-like cells. Likewise, cells cultured on hydroxyapatite + chitosan had the highest rate of osteonectin mRNA expression over two weeks, indicating enhanced osteoinduction exerted by the composite. The role of composite scaffolds containing chitosan on periosteal cell behavior (*i.e.*, on bone tissue regeneration) was strengthened in the work by Gentile *et al.* [196]. Therefore, Frohberg *et al.* proposed that these scaffolds might be useful for repair of maxillofacial defects and injuries (Figure 8) [197]. In fact, films crosslinked with genipin significantly promoted adhesion, proliferation, and differentiation of pre-osteoblasts.

Resin-based sealants containing chitosan nanofibers were developed by Mahapoka *et al.* and reviewed by Li XM *et al.* [198,199]. Chitosan whiskers were incorporated into dimethacrylate monomer at various ratios by weight. The dimethacrylate-based sealant containing chitosan nanofibers had a greater antimicrobial activity than control sealant and they were comparable with commercial sealants. The inclusion of the whiskers did not reduce the curing depth, and the reduction in hardness was minimal. In conclusion, resin-based sealants containing chitosan nanofibers are effective antimicrobial pit and fissure sealants with enhanced performances as dental restorative materials.

### 5.3. Bacteria, Fungi, and Viruses

By using Na hypochlorite, Dutta *et al.* substituted the hydrogen atom in the N-H group of chitin with a chlorine atom, to generate the N-Cl bond on the n-chitin surface, while maintaining the characteristic n-chitin morphology [200]. Although plain chitosan can be *N*-chlorinated [201], there is interest in preferring n-chitin because the latter is stably dispersed in water, and has large surface area, excellent mechanical properties, and prompt filmogenicity, as points of superiority compared with plain chitosan. The antimicrobial properties of *N*-chlorine stem from the direct transfer of the oxidative chlorine from the n-chitin films to the most susceptible parts of the microbes, thus effectively dismantling or inhibiting their enzymatic or metabolic processes.

Thus, n-chitin in *N*-halamine form kills microorganisms without the release of free chlorine. Upon depletion, the chloramine function is rechargeable with another NaClO treatment. Table 1 shows some data of bacteriological interest. Moreover, the films exerted 100% and 80% inhibition of fungal spore germination against *Alternaria alternata* and *Penicillium digitatum*, respectively [200]. *N*-(2-Hydroxy-3-trimethylammonium)propyl chitosan and *N*-benzyl-*N*,*N*-dimethyl chitosan were prepared in the form of e-spun mats. Both *S. aureus* and *E. coli* did not survive after 4 h contact with each of them [202].

**Table 1:** Percentage reduction of *E. coli* and *S. aureus* by chlorinated nano-chitin films [200].

Contact Time, Min	*Escherichia coli*, %	*Staphylococcus aureus*, %
5	86.4	46.7
10	99.9	87.8
30	100.0	100.0
60	100.0	100.0

Bacterial concentrations were 108–109 CFU/mL. The chlorinated n-chitin film contained 2.69% of active chlorine. The non chlorinated chitosan did not affect the bacteria. Cellulose membranes were used as negative control.

As for viruses, the *N*-(2-hydroxy-3-trimethylammonium)propyl chitosan was e-spun into nanofibers with PVA, to yield a mat with a large surface area that is a major requirement for effective adsorption. The nanofibers were crosslinked with glutaraldehyde to impart stability in aqueous media, thus the swelling of the fibers after 6 h in water was limited to 30%. Tests for their capacity to remove the non-enveloped porcine parvovirus and the enveloped Sindbis virus yielded values that came close to or exceeded the EPA regulation values for virus removal processes [203].

## 6. Conclusions and Perspectives

The above study of the current literature indicates that the nano size enhances the performances of chitins and chitosans, with no exceptions, ranging from human tissue regeneration to microbicidal activity; the most impressive data being those indicating that n-chitin alleviates the clinical symptoms and suppresses the onset of ulcerative colitis in an animal model.

The chitin nanofibrils are today isolated by quite simple mechanical means, that do not present scaling-up problems. It should be remarked however that for n-chitin itself and especially for n-chitosan, the bottleneck seems to reside in the deacetylation of the raw chitin. In particular the deacetylation is always carried out with the aid of boiling NaOH, which can be a dangerous and expensive operation.

A totally different panorama is offered by e-spinning. Although e-spun materials have been available for a long time, and the technique has been claimed to be versatile, their structure is either fluffy or offers too small pores, so that cellular infiltration is precluded. Moreover, e-spinning is based exclusively on ejection from a needle at extremely low speed, and scaling-up is quite a problem. New approaches such as force-spinning might be developed as described by Lozano *et al.* but it seems that there is still a long way to go to take e-spun materials to the clinical use [204]. On the other hand, e-spinning is a powerful stimulus for doing basic research on nano materials.

Turning to realistic perspectives, it would seem that biotechnological approaches are available immediately to modify certain situations that delay the exploitation of the chitin resources. For the sake of clarity let us consider some examples. (**a**) The study of the enzymatic deacetylation of n-chitin has not been undertaken so far, notwithstanding the fact that engineered deacetylases are available today [205]. No information exists on the behavior of deacetylases on n-chitin; (**b**) *Serratia marcescens* B742 mutants were prepared to improve the deproteination of shrimp shell powders, in fact 91.4% was achieved after three days of fermentation [206]; (**c**) Yeast spores can be deprived of their outermost dityrosine layer by genetic engineering, thus exposing their chitosan layer which becomes available for collection of metals, enzymes, sterols, and for use in medication [207]; (**d**) Certain agroindustrial discards (corn steep liquor and molasses) can be converted into chitosan by *Rhizopus arrhizus* and *Cunninghamella elegans* [208]; (**e**) The fisheries themselves have much to gain by using n-chitin and n-chitosan for the improved preservation of crustaceans [209]; (**f**) The treatment of fresh by-products from the canning factories should be revised in the light of existing advanced technologies [210].

The production of medical-grade chitosan took many years of effort in coordinating the activities of the fisheries with those of industries and research laboratories for quality assessment and certification. The currently marketed medical-grade chitosan derives from purified chitins that are also suitable for the isolation of n-chitin: for n-chitin, therefore, the development stage might be shorter, but it demands the same endeavor.

## References

[1] Muzzarelli R.A.A. (2011). Chitin nanostructures in living organisms. Chitin Formation and Diagenesis.

[2] Muzzarelli R.A.A. (2012). Nanochitins and nanochitosans, paving the way to eco-friendly and energy-saving exploitation of marine resources. Polym. Sci. Compr. Ref..

[3] Muzzarelli R.A.A., Boudrant J., Meyer D., Manno N., DeMarchis M., Paoletti M.G. (2012). A tribute to Henri Braconnot, precursor of the carbohydrate polymers science, on the chitin bicentennial. Carbohydr. Polym..

[4] Muzzarelli R.A.A., Muzzarelli C., Dutta P.K. (2005). Chitin and Chitosan: Opportunities and Challenges.

[5] Muzzarelli R.A.A. (2011). Biomedical exploitation of chitin and chitosan via mechano-chemical disassembly, electrospinning, dissolution in imidazolium ionic liquids, and supercritical drying. Mar. Drugs.

[6] Muzzarelli R.A.A., Jayakumar R., Prabaharan A., Muzzarelli R.A.A. (2011). New techniques for optimization of surface area and porosity in nanochitins and nanochitosans. Advances in Polymer Science: Chitosan for Biomaterials.

[7] Muzzarelli R.A.A. (1977). Chitin.

[8] Araki J. (2013). Electrostatic or steric? Preparations and characterizations of well-dispersed systems containing rod-like nanowhiskers of crystalline polysaccharides. Soft Matter.

[9] Ifuku S., Nomura R., Morimoto M., Saimoto H. (2011). Preparation of chitin nanofibers from mushrooms. Materials.

[10] Ifuku S., Nogi M., Abe K., Yoshioka M., Morimoto M., Saimoto H., Yano H. (2011). Simple preparation of chitin nanofibers with a width of 10–20 nm from prawn shell under neutral conditions. Carbohydr. Polym..

[11] Ifuku S., Saimoto H. (2012). Chitin nanofibers: Preparations, modifications, and applications. Nanoscale.

[12] Chatrabhuti S., Chirachanchai S. (2013). Single step coupling for multi-responsive water-based chitin/chitosan magnetic nanoparticles. Carbohydr. Polym..

[13] Sahay R., Kumar P.S., Sridhar R., Sundaramurthy J., Venugopal J., Mhaisalkar S.G., Ramakrishna S. (2012). Electrospun composite nanofibers and their multifaceted applications. J. Mater. Chem..

[14] Neville A.C. (1993). Biology of Fibrous Composites: Development beyond the Cell Membrane.

[15] Muzzarelli R.A.A., Jeuniaux C., Gooday G.W. (1986). Chitin in Nature and Technology.

[16] Stankiewicz B.A., van Bergen P. (1998). Nitrogen-Containing Macromolecules in the Bio- and Geosphere.

[17] Jollès P., Muzzarelli R.A.A. (1999). Chitin and Chitinases.

[18] Kurita K. (2006). Chitin and chitosan: Functional biopolymers from marine crustaceans. Mar. Biotechnol..

[19] Ravi Kumar M.N.V., Muzzarelli R.A.A., Muzzarelli C., Sashiwa H., Domb A.J. (2004). Chitosan chemistry and pharmaceutical perspectives. Chem. Rev..

[20] Keong L.C., Halim A.S. (2009). *In vitro* models in biocompatibility assessment for biomedical-grade chitosan derivatives in wound management (Review). Int. J. Mol. Sci..

[21] Desbrieres J., Babak V.G. (2008). Interfacial properties of amphiphilic systems on the basis of natural polymers-chitin derivatives. Russ. J. Gen. Chem..

[22] Muzzarelli R.A.A. (2010). Chitins and chitosans as immunoadjuvants and non-allergenic drug carriers. Mar. Drugs.

[23] Grunenfelder L.K., Herrera S., Kisailus D. (2014). Crustacean-derived biomimetic components and nanostructured composites. Small.

[24] Yan W.X., Shen L.B., Ji Y.L., Yang Q., Shen X.Y. (2014). Chitin nanocrystal reinforced wet-spun chitosan fibers. J. Appl. Polym. Sci..

[25] Sashiwa H., Aiba S.I. (2004). Chemically modified chitin and chitosan as biomaterials. Prog. Polym. Sci..

[26] Gomez d’Ayala G., Malinconico M., Laurienzo P. (2008). Marine derived polysaccharides for biomedical applications: Chemical modification approaches. Molecules.

[27] Mincea M., Negrulescu A., Ostafe V. (2012). Preparation, modification, and applications of chitin nanowhiskers: A review. Rev. Adv. Mater. Sci..

[28] Cortizo M.S., Berghoff C.F., Alessandrini J.L. (2008). Characterization of chitin from *Illex argentinus* squid pen. Carbohydr. Polym..

[29] Yui T., Taki N., Sugiyama J., Hayashi S. (2007). Exhaustive crystal structure search and crystal modeling of beta-chitin. Int. J. Biol. Macromol..

[30] Lavall R.L., Assis O.B.G., Campana S.P. (2007). Beta-Chitin from the pens of *Loligo* sp.: Extraction and characterization. Bioresour. Technol..

[31] Chandumpai A., Singhpibulporn N., Faroongsarng D., Sornprasit P. (2004). Preparation and physico-chemical characterization of chitin and chitosan from the pens of the squid species, *Loligo lessoniana* and *Loligo formosana*. Carbohydr. Polym..

[32] Nishiyama Y., Noishiki Y., Wada M. (2011). X-ray Structure of Anhydrous beta-Chitin at 1 angstrom Resolution. Macromolecules.

[33] Sawada D., Nishiyama Y., Langan P., Forsyth V.T., Kimura S., Wada M. (2012). Water in crystalline fibers of dihydrate beta-chitin results in unexpected absence of intramolecular hydrogen bonding. PLoS One.

[34] Yang F.C., Peters R.D., Dies H., Rheinstadter M.C. (2014). Hierarchical, self-similar structure in native squid pen. Soft Matter.

[35] Sawada D., Ogawa Y., Kimura S., Nishiyama Y., Langan P., Wada M. (2014). Solid-solvent molecular interactions observed in crystal structures of beta-chitin complexes. Cellulose.

[36] Youn D.K., No H.K., Prinyawiwatkul W. (2013). Preparation and characteristics of squid pen beta-chitin prepared under optimal deproteination and demineralisation condition. Int. J. Food Sci. Technol..

[37] Fabritius H., Sachs C., Raabe D., Nikolov S., Friak M., Neugebauer J. (2011). Chitin in the exoskeletons of arthropoda: From ancient design to novel materials science. Chitin Formation and Diagenesis: 34.

[38] Fabritius H.O., Karsten E.S., Balasundaram K., Hild S., Huemer K., Raabe D. (2012). Correlation of structure, composition and local mechanical properties in the dorsal carapace of the edible crab *Cancer pagurus*. Zeitschrift Fur Kristallographie.

[39] Raabe D., Al-Sawalmih A., Romano P., Sachs C., Brokmeier H.G., Yi S.B., Servos G., Hartwig H.G. (2005). Structure and crystallographic texture of arthropod bio-composites. Icotom 14: Texture Materi..

[40] Raabe D., Romano P., Sachs C. (2005). The crustacean exoskeleton as an example of a structurally and mechanically graded biological nanocomposite material. Acta Mater..

[41] Raabe D., Romano P., Sachs C., Al-Sawalmih A., Brokmeier H.G., Yi S.B., Servos G., Hartwig H.G. (2005). Discovery of a honeycomb structure in the twisted plywood patterns of fibrous biological nanocomposite tissue. J. Cryst. Growth.

[42] Raabe D., Romano P., Sachs C., Fabritius H., Al-Sawalmih A., Yi S.B., Servos G., Hartwig H.G. (2006). Microstructure and crystallographic texture of the chitin-protein network in the biological composite material of the exoskeleton of the lobster *Homarus americanus*. Mater. Sci. Eng..

[43] Raabe D., Al-Sawalmih A., Yi S.B., Fabritius H. (2007). Preferred crystallographic texture of alpha-chitin as a microscopic and macroscopic design principle of the exoskeleton of the lobster *Homarus americanus*. Acta Biomater..

[44] Nikolov S., Fabritius H., Petrov M., Friak M., Lymperakis L., Sachs C., Raabe D., Neugebauer J. (2011). Robustness and optimal use of design principles of arthropod exoskeletons studied by ab initio-based multiscale simulations. J. Mech. Behav. Biomed. Mater..

[45] Raue L., Klein H., Raabe D. (2010). The exoskeleton of the american lobster: From texture to anisotropic properties. Texture Anisotropy Polycrystals III.

[46] Saito Y., Okano T., Chanzy H., Sugiyama J. (1995). Structural study of alpha-chitin from the grasping spines of the arrow worm *Sagitta* spp.. J. Struct. Biol..

[47] Bone Q., Ryan K., Pulsford A.L. (1983). The structure and the composition of the teeth and grasping spines of Chaetognaths. J. Mar. Biol. Assoc. UK.

[48] Chretiennot-Dinet M.J., Giraud-Guille M.M., Vaulot D., Putaux J.L., Saito Y., Chanzy H. (1997). The chitinous nature of filaments ejected by *Phaeocystis* (Prymnesiophyceae). J. Phycol..

[49] Rousseau V., Lantoine F., Rodriguez F., LeGall F., Chretiennot-Dinet M.J., Lancelot C. (2013). Characterization of *Phaeocystis globosa* (Prymnesiophyceae), the blooming species in the Southern North Sea. J. Sea Res..

[50] Barbosa S.S., Kelaher B.P., Byrne M. (2010). Patterns of abundance, growth and size of the tropical intertidal chiton *Acanthopleura gemmate*. Molluscan Res..

[51] Kelly R.P., Eernisse D.J. (2008). Reconstructing a radiation: The chiton genus Mopalia in the north Pacific. Invertebr. Syst..

[52] Evans L.A., Macey D.J., Webb J. (1990). Characterization and structural organization of the organic matrix of the radula teeth of the chiton *Acanthopleura hirtosa*. Philos. Trans. R. Soc. Lond. B.

[53] Shaw J.A., Macey D.J., Brooker L.R. (2008). Radula synthesis by three species of iron mineralizing molluscs: Production rate and elemental demand. J. Mar. Biol. Assoc. UK.

[54] Wang Q.Q., Nemoto M., Li D.S., Weaver J.C., Weden B., Stegemeier J., Bozhilov K.N., Wood L.R., Milliron G.W., Kim C.S. (2013). Phase transformations and structural developments in the radular teeth of *Cryptochiton stelleri*. Adv. Funct. Mater..

[55] Minke R., Blackwell J. (1978). The structure of alpha chitin. J. Mol. Biol..

[56] Noishiki Y., Nishiyama Y., Wada M., Okada S., Kuga S. (2003). Inclusion complex of beta-chitin and aliphatic amines. Biomacromolecules.

[57] Miserez A., Li Y.L., Waite J.H., Zok F. (2007). Jumbo squid beaks: Inspiration for design of robust organic composites. Acta Biomater..

[58] Muzzarelli R.A.A., Muzzarelli C., Cosani A., Terbojevich M. (1999). 6-Oxychitins, novel hyaluronan-like regiospecifically carboxylated chitins. Carbohydr. Polym..

[59] Lai C., Zhang S.J., Chen X.C., Sheng L.Y. (2014). Nanocomposite films based on TEMPO-mediated oxidized bacterial cellulose and chitosan. Cellulose.

[60] Fan Y.M., Fukuzumi H., Saito T., Isogai A. (2012). Comparative characterization of aqueous dispersions and cast films of different chitin nanowhiskers/nanofibers. Int. J. Biol. Macromol..

[61] Zhou J., Butchosa N., Jayawardena H.S.N., Zhou Q., Yan M.D., Ramstrom O. (2014). Glycan-functionalized fluorescent chitin nanocrystals for biorecognition applications. Bioconjug. Chem..

[62] Fan Y.M., Saito T., Isogai A. (2008). Preparation of chitin nanofibers from squid pen beta-chitin by simple mechanical treatment under acid conditions. Biomacromolecules.

[63] Fan Y.M., Saito T., Isogai A. (2008). Chitin nanocrystals prepared by TEMPO-mediated oxidation of alpha-chitin. Biomacromolecules.

[64] Dutta A.K., Yamada K., Izawa H., Morimoto M., Saimoto H., Ifuku S. (2013). Preparation of chitin nanofibers from dry chitin powder by star burst system: Dependence on number of passes. J. Chitin Chitosan Sci..

[65] Ifuku S., Nogi M., Abe K., Yoshioka M., Morimoto M., Saimoto H., Yano H. (2009). Preparation of chitin nanofibers with a uniform width as alpha-chitin from crab shells. Biomacromolecules.

[66] Fan Y.M., Saito T., Isogai A. (2010). Individual chitin nano-whiskers prepared from partially deacetylated alpha-chitin by fibril surface cationization. Carbohydr. Polym..

[67] Abe K., Ifuku S., Kawata M., Yano H. (2014). Preparation of tough hydrogels based on beta-chitin nanofibers via NaOH treatment. Cellulose.

[68] Mushi N.E., Butchosa N., Salajkova M., Zhoua Q., Berglund L.A. (2014). Nanostructured membranes based on native chitin nanofibers prepared by mild process. Carbohydr. Polym..

[69] Wijesena R., Tissera N., Kannankgara Y., Lin Y., Amaratunga G., de Silva N. (2014). A method for top down preparation of chitosan nanoparticles and nanofibers. Carbohydr. Polym..

[70] Watthanaphanit A., Supaphol P., Tamura H., Tokura S., Rujiravanit R. (2010). Wet-spun alginate/chitosan whiskers nanocomposite fibers: Preparation, characterization and release characteristic of the whiskers. Carbohydr. Polym..

[71] Ifuku S., Ikuta A., Egusa M., Kaminaka H., Izawa H., Morimoto M., Saimoto H. (2013). Preparation of high-strength transparent chitosan film reinforced with surface-deacetylated chitin nanofibers. Carbohydr. Polym..

[72] Kadokawa J., Takegawa A., Mine S., Prasad K. (2011). Preparation of chitin nanowhiskers using an ionic liquid and their composite materials with poly(vinyl alcohol). Carbohydr. Polym..

[73] Liu D.G., Wu Q.L., Chang P.R., Gao G.Z. (2011). Self-assembled liquid crystal film from mechanically defibrillated chitosan nanofibers. Carbohydr. Polym..

[74] Saito Y., Putaux J.L., Okano T., Gaill F., Chanzy H. (1997). Structural aspects of the swelling of beta chitin in HCl and its conversion into alpha chitin. Macromolecules.

[75] Jin J., Hassanzadeh P., Perotto G., Sun W., Brenckle M.A., Kaplan D., Omenetto F.G., Rolandi M. (2013). A biomimetic composite from solution self-assembly of chitin nanofibers in a silk fibroin matrix. Adv. Mater..

[76] Zhong C., Kapetanovic A., Deng Y.X., Rolandi M. (2011). A chitin nanofiber ink for airbrushing, replica molding, and microcontact printing of self-assembled macro-, micro-, and nanostructures. Adv. Mater..

[77] Rolandi M., Rolandi R. (2014). Self-assembled chitin nanofibers and applications. Adv. Colloid Interface Sci..

[78] Hassanzadeh P., Kharaziha M., Nikkhah M., Shin S.R., Jin J., He S., Sun W., Zhong C., Dokmeci M.R., Khademhosseini A. (2013). Chitin nanofiber micropatterned flexible substrates for tissue engineering. J. Mater. Chem. B.

[79] Fuh Y.K., Chen S.Z., Jang J.S.C. (2012). Direct-write, well-aligned chitosan-poly(ethylene oxide) nanofibers deposited via near-field electrospinning. J. Macromol. Sci. A.

[80] Cooper A., Zhong C., Kinoshita Y., Morrison R.S., Rolandi M., Zhang M.Q. (2012). Self-assembled chitin nanofiber templates for artificial neural networks. J. Mater. Chem..

[81] Wu J., Meredith J.C. (2014). Assembly of chitin nanofibers into porous biomimetic structures via freeze drying. Acs Macro Lett..

[82] Wu Y., Sasaki T., Irie S., Sakurai K. (2008). A novel biomass-ionic liquid platform for the utilization of native chitin. Polymer.

[83] Hu X.W., Du Y.M., Tang Y.F., Wang Q., Feng T., Yang J.H., Kennedy J.F. (2007). Solubility and property of chitin in NaOH/urea aqueous solution. Carbohydr. Polym..

[84] Hu X.W., Tang Y.F., Wang Q., Li Y., Yang J.H., Du Y.M., Kennedy J.F. (2011). Rheological behaviour of chitin in NaOH/urea aqueous solution. Carbohydr. Polym..

[85] Huang Y., Zhong Z.B., Duan B., Zhang L.N., Yang Z.X., Wang Y.F., Ye Q.F. (2014). Novel fibers fabricated directly from chitin solution and their application as wound dressing. J. Mater. Chem. B.

[86] Li G.X., Du Y.M., Tao Y.Z., Liu Y.T., Li S., Hu X.W., Yang J. (2010). Dilute solution properties of four natural chitin in NaOH/urea aqueous system. Carbohydr. Polym..

[87] Nata I.F., Wang S.S.S., Wu T.M., Lee C.K. (2012). β-Chitin nanofibrils for self-sustaining hydrogels preparation via hydrothermal treatment. Carbohydr. Polym..

[88] Ifuku S., Morooka S., Morimoto M., Saimoto H. (2010). Acetylation of chitin nanofibers and their transparent nanocomposite films. Biomacromolecules.

[89] Ifuku S., Iwasaki M., Morimoto M., Saimoto H. (2012). Graft polymerization of acrylic acid onto chitin nanofiber to improve dispersibility in basic water. Carbohydr. Polym..

[90] Chen C.C., Li D.G., Deng Q.Y., Zheng B.T. (2012). Optically transparent biocomposites: Polymethylmethacrylate reinforced with high-performance chitin nanofibers. Bioresources.

[91] Chen C.C., Li D.G., Hu Q.Q., Wang R. (2014). Properties of polymethyl methacrylate-based nanocomposites: Reinforced with ultra-long chitin nanofiber extracted from crab shells. Mater. Des..

[92] Ifuku S., Ikuta A., Izawa H., Morimoto M., Saimoto H. (2014). Control of mechanical properties of chitin nanofiber film using glycerol without losing its characteristics. Carbohydr. Polym..

[93] Shams M.I., Yano H. (2013). Simplified fabrication of optically transparent composites reinforced with nanostructured chitin. J. Polym. Environ..

[94] Lu Y., Sun Q.F., She X.L., Xia Y.Z., Liu Y.X., Li J., Yang D.J. (2013). Fabrication and characterisation of α-chitin nanofibers and highly transparent chitin films by pulsed ultrasonication. Carbohydr. Polym..

[95] Huang Y.C., Wu Y., Huang W.C., Yang F., Ren X.E. (2013). Degradation of chitosan by hydrodynamic cavitation. Polymer. Degrad. Stab..

[96] Gao Y., Truong Y.B., Zhu Y.G., Kyratzis I.L. (2014). Electrospun antibacterial nanofibers: Production, activity, and *in vivo* applications. J. Appl. Polym. Sci..

[97] Goh Y.F., Shakir I., Hussain R. (2013). Electrospun fibers for tissue engineering, drug delivery and wound dressing. J. Mater. Sci..

[98] Jayakumar R., Prabaharan M., Nair S.V., Tamura H. (2010). Novel chitin and chitosan nanofibers in biomedical applications. Biotechnol. Adv..

[99] Rosic R., Pelipenko J., Kocbek P., Baumgartner S., Bester-Rogac M., Kristl J. (2012). The role of rheology of polymer solutions in formation by electrospinning. Eur. Polym. J..

[100] Wang X.F., Ding B., Sun G., Wang M.R., Yu J.Y. (2013). Electro-spinning/netting: A strategy for the fabrication of three-dimensional polymer nano-fiber/nets. Prog. Mater. Sci..

[101] Junkasem J., Rujiravanit R., Supaphol P. (2006). Fabrication of alpha-chitin whisker-reinforced poly(vinyl alcohol) nanocomposite nanofibres by electrospinning. Nanotechnology.

[102] Junkasem J., Rujiravanit R., Grady B.P., Supaphol P. (2010). X-ray diffraction and dynamic mechanical analyses of alpha-chitin whisker-reinforced poly(vinyl alcohol) nanocomposite nanofibers. Polym. Int..

[103] Austin P.R., Zikakis J.P. (1984). Chitin solvents and solubility parameters. Chitin, Chitosan, and Related Enzymes.

[104] Austin P.R., Brine C.J., Castle J.E., Zikakis J.P. (1981). Chitin: New facets of research. Science.

[105] Park K.E., Kang H.K., Lee S.J., Min B.M., Park W.H. (2006). Biomimetic nanofibrous scaffolds: Preparation and characterization of PGA/chitin blend nanofibers. Biomacromolecules.

[106] Shalumon K.T., Binulal N.S., Selvamurugan N., Nair S.V., Menon D., Furuike T., Tamura H., Jayakumar R. (2009). Electrospinning of carboxymethyl chitin/poly(vinyl alcohol) nanofibrous scaffolds for tissue engineering applications. Carbohydr. Polym..

[107] Heath L., Zhu L.F., Thielemans W. (2013). Chitin nanowhisker aerogels. Chemsuschem.

[108] Ding B.B., Cai J., Huang J.C., Zhang L.N., Chen Y., Shi X.W., Du Y.M., Kuga S. (2012). Facile preparation of robust and biocompatible chitin aerogels. J. Mater. Chem..

[109] Elsabee M.Z., Naguib H.F., Morsi R.E. (2012). Chitosan based nanofibers, a review. Mater. Sci. Eng. C-Mater. Biol. Appl..

[110] Mucha M., Balcerzak J., Michalak I., Tylman M. (2011). Biopolymeric matrices based on chitosan for medical applications. E-Polymers.

[111] Nirmala R., Il B.W., Navamathavan R., El-Newehy M.H., Kim H.Y. (2011). Preparation and characterizations of anisotropic chitosan nanofibers via electrospinning. Macromol. Res..

[112] Ohkawa K., Cha D.I., Kim H., Nishida A., Yamamoto H. (2004). Electrospinning of chitosan. Macromol. Rapid Commun..

[113] Dilamian M., Montazer M., Masoumi J. (2013). Antimicrobial electrospun membranes of chitosan/poly(ethylene oxide) incorporating poly(hexamethylene biguanide) hydrochloride. Carbohydr. Polym..

[114] Hu W.W., Yu H.N. (2013). Co-electrospinning of chitosan/alginate fibers by dual-jet system for modulating material surfaces. Carbohydr. Polym..

[115] Hu W., Huang Z.M. (2010). Biocompatibility of braided poly(l-lactic acid) nanofiber wires applied as tissue sutures. Polym. Int..

[116] Li Y.J., Chen F., Nie J., Yang D.Z. (2012). Electrospun poly(lactic acid)/chitosan core-shell structure nanofibers from homogeneous solution. Carbohydr. Polym..

[117] Mathew A.P., Laborie M.P.G., Oksman K. (2009). Cross-linked chitosan/chitin crystal nanocomposites with improved permeation selectivity and pH stability. Biomacromolecules.

[118] Min B.M., Lee S.W., Lim J.N., You Y., Lee T.S., Kang P.H., Park W.H. (2004). Chitin and chitosan nanofibers: Electrospinning of chitin and deacetylation of chitin nanofibers. Polymer.

[119] Pereira A.G.B., Muniz E.C., Hsieh Y.L. (2014). Chitosan-sheath and chitin-core nanowhiskers. Carbohydr. Polym..

[120] Sangsanoh P., Supaphol P. (2006). Stability improvement of electrospun chitosan nanofibrous membranes in neutral or weak basic aqueous solutions. Biomacromolecules.

[121] Nam Y.S., Park W.H., Ihm D., Hudson S.M. (2010). Effect of the degree of deacetylation on the thermal decomposition of chitin and chitosan nanofibers. Carbohydr. Polym..

[122] Torres-Giner S., Ocio M.J., Lagaron J.M. (2008). Development of active antimicrobial fiber based chitosan polysaccharide nanostructures using electrospinning. Eng. Life Sci..

[123] Geng X.Y., Kwon O.H., Jang J.H. (2005). Electrospinning of chitosan dissolved in concentrated acetic acid solution. Biomaterials.

[124] Shalumon K.T., Anulekha K.H., Girish C.M., Prasanth R., Nair S.V., Jayakumar R. (2010). Single step electrospinning of chitosan/poly(caprolactone) nanofibers using formic acid/acetone solvent mixture. Carbohydr. Polym..

[125] Ji Y.L., Liang K., Shen X.Y., Bowlin G.L. (2013). Electrospinning and characterization of chitin nanofibril/polycaprolactone nanocomposite fiber mats. Carbohydr. Polym..

[126] Bhattarai N., Edmondson D., Veiseh O., Matsen F.A., Zhang M. (2005). Electrospun chitosan-based nanofibers and their cellular compatibility. Biomaterials.

[127] Lee K.H., Shin S.J., Kim C.B., Kim J.K., Cho Y.W., Chung B.G., Lee S.H. (2010). Microfluidic synthesis of pure chitosan microfibers for bio-artificial liver chip. Lab Chip.

[128] Casettari L., Cespi M., Castagnino E. (2012). Evaluation of dibutyrylchitin as new excipient for sustained drug release. Drug Dev. Ind. Pharm..

[129] Castagnino E., Ottaviani M.F., Cangiotti M., Morelli M., Casettari L., Muzzarelli R.A.A. (2008). Radical scavenging activity of 5-methylpyrrolidinone chitosan and dibutyryl chitin. Carbohydr. Polym..

[130] Jeon I.H., Mok J.Y., Park K.H., Hwang H.M., Song M.S., Lee D., Lee M.H., Lee W.Y., Chai K.Y., Jang S.I. (2012). Inhibitory effect of dibutyryl chitin ester on nitric oxide and prostaglandin E-2 production in LPS-stimulated RAW 264.7 cells. Arch. Pharm. Res..

[131] Muzzarelli C., Francescangeli O., Tosi G., Muzzarelli R.A.A. (2004). Susceptibility of dibutyryl chitin and regenerated chitin fibres to deacylation and depolymerization by lipases. Carbohydr. Polym..

[132] Muzzarelli R.A.A., Guerrieri M., Goteri G., Muzzarelli C., Armeni T., Ghiselli R., Cornelissen M. (2005). The biocompatibility of dibutyryl chitin, in the context of wound dressings. Biomaterials.

[133] Bogun M., Krucinska I., Kommisarczyk A., Mikolajczyk T., Blazewicz M., Stodolak-Zych E., Menaszek E., Scislowska-Czarnecka A. (2013). Fibrous polymeric composites based on alginate fibres and fibres made of poly-epsilon-caprolactone and dibutyryl chitin for use in regenerative medicine. Molecules.

[134] Jang S.I., Mok J.Y., Jeon I.H., Park K.H., Thuy T.T.N., Park J.S., Hwang H.M., Song M.S., Lee D., Chai K.Y. (2012). Effect of electrospun non-woven mats of dibutyryl chitin/poly(lactic acid) blends on wound healing in hairless mice. Molecules.

[135] Schoukens G. (2009). Bioactive dressings to promote wound healing. Adv. Text. Wound Care.

[136] Azuma K., Ifuku S., Osaki T., Okamoto Y., Minami S. (2014). Preparation and biomedical applications of chitin and chitosan nanofibers. J. Biomed. Nanotechnol..

[137] Ding F.Y., Deng H.B., Du Y.M., Shi X.W., Wang Q. (2014). Emerging chitin and chitosan nanofibrous materials for biomedical applications. Nanoscale.

[138] Jin S.B., Li S.L., Wang C.X., Liu J., Yang X.L., Wang P.C., Zhang X., Liang X.J. (2014). Biosafe nanoscale pharmaceutical adjuvant materials. J. Biomed. Nanotechnol..

[139] Singh D., Han S.S., Shin E.J. (2014). Polysaccharides as nanocarriers for therapeutic applications. J. Biomed. Nanotechnol..

[140] DiLena F. (2014). Hemostatic polymers: The concept, state of the art and perspectives. J. Mater. Chem. B.

[141] Muzzarelli R.A.A. (1993). Biochemical significance of exogenous chitins and chitosans in animals and patients. Carbohydr. Polym..

[142] Kelechi T.J., Mueller M., Hankin C.S., Bronstone A., Samies J., Bonham P.A. (2012). A randomized, investigator-blinded, controlled pilot study to evaluate the safety and efficacy of a poly-*N*-acetyl glucosamine-derived membrane material in patients with venous leg ulcers. J. Am. Acad. Dermatol..

[143] Fischer T.H., Hays W.E., Valeri C.R. (2011). Poly-*N*-acetyl glucosamine fibers accelerate hemostasis in patients treated with antiplatelet drugs. J. Trauma-Inj. Infect. Crit. Care.

[144] Lindner H.B., Zhang A.G., Eldridge J., Demcheva M., Tsichilis P., Seth A., Vournakis J., Muise-Helmericks R.C. (2011). Anti-bacterial effects of poly-*N*-acetyl-glucosamine nanofibers in cutaneous wound healing: Requirement for Akt1. PLoS One.

[145] Blanco-Padilla A., Soto K.M., Iturriaga M.H., Mendoza S. (2014). Food antimicrobials nanocarriers. Sci. World J..

[146] Busilacchi A., Gigante A., Mattioli-Belmonte M., Muzzarelli R.A.A. (2013). Chitosan stabilizes platelet growth factors and modulates stem cell differentiation toward tissue regeneration. Carbohydr. Polym..

[147] Scherer S.S., Pietramaggiori G., Matthews J.C., Gennaoui A., Demcheva M., Fischer T.H., Valeri C.R., Orgill D.P. (2011). Poly-*N*-acetyl glucosamine fibers induce angiogenesis in ADP inhibitor-treated diabetic mice. J. Trauma-Inj. Infect. Crit. Care.

[148] Erba P., Adini A., Demcheva M., Valeri C.R., Orgill D.P. (2011). Poly-*N*-acetyl glucosamine fibers are synergistic with vacuum-assisted closure in augmenting the healing response of diabetic mice. J. Trauma-Inj. Infect. Crit. Care.

[149] Gorapalli D., Seth A., Vournakis J., Whyne C., Akens M., Zhang A.G., Demcheva M., Qamirani E., Yee A. (2012). Evaluation of a novel poly *N*-acetyl glucosamine (pGlcNAc) hydrogel for treatment of the degenerating intervertebral disc. Life Sci..

[150] Muise-Helmericks R.C., Demcheva M., Vournakis J.N., Seth A. (2011). Poly-*N*-acetyl glucosamine fibers activate bone regeneration in a rabbit femur injury model. J. Trauma-Inj. Infect. Crit. Care.

[151] Muzzarelli R.A.A. (2009). Chitins and chitosans for the repair of wounded skin, nerve, cartilage and bone. Carbohydr. Polym..

[152] Muzzarelli R.A.A., Mattioli-Belmonte M., Tietz C., Brunelli M.A., Fini M., Giardino R., Ilari P., Biagini G. (1994). Stimulatory effect on bone formation exerted by a modified chitosan. Biomaterials.

[153] Mattioli-Belmonte M., Nicoli-Aldini N., DeBenedittis A., Sgarbi G., Amati S., Fini M., Biagini G., Muzzarelli R.A.A. (1999). Morphological study of bone regeneration in the presence of 6-oxychitin. Carbohydr. Polym..

[154] Malho J.M., Heinonen H., Kontro I., Mushi N.E., Serimaa R., Hentze H.P., Linder M.B., Szilvay G.R. (2014). Formation of ceramophilic chitin and biohybrid materials enabled by a genetically engineered bifunctional protein. Chem. Commun..

[155] Nakayama S., Suzuki M., Endo H., Iimura K., Kinoshita S., Watabe S., Kogure T., Nagasawa H. (2013). Identification and characterization of a matrix protein in the periostracum of the pearl oyster, Pinctada fucata. FEBS Open Biol..

[156] Azuma K., Osaki T., Wakuda T., Ifuku S., Saimoto H., Tsuka T., Imagawa T., Okamoto Y., Minami S. (2012). Beneficial and preventive effect of chitin nanofibrils in a dextran sulfate sodium-induced acute ulcerative colitis model. Carbohydr. Polym..

[157] Baker D.E., Kane S. (2004). The short and long-term safety of 5-aminosalicylate products in the treatment of ulcerative colitis. Rev. Gastroenterol. Disord..

[158] Kane S., Bjorkman D.J. (2013). The efficacy of oral 5-ASA in the treatment of active ulcerative colitis: A systematic review. Rev. Gastroenterol. Disord..

[159] Lowry P.W., Franklin C.L., Weaver A.L., Szumlanski C.L., Mays D.C., Loftus E.V., Tremaine W.J., Lipsky J.J., Weinshilboum R.M., Sandborn W.J. (2001). Leucopenia resulting from a drug interaction between azathioprine or 6-mercaptopurine and mesalamine, sulphasalazine or balsalazide. Gut.

[160] Azuma K., Osaki T., Ifuku S., Saimoto H., Tsuka T., Imagawa T., Okamoto Y., Minami S. (2012). α-Chitin nanofibrils improve inflammatory and fibrosis responses in inflammatory bowel disease mice model. Carbohydr. Polym..

[161] Muzzarelli R.A.A., Morganti P., Morganti G., Palombo P., Palombo M., Biagini G., Belmonte M.M., Giantomassi F., Orlandi F., Muzzarelli C. (2007). Chitin nanofibrils/chitosan glycolate composites as wound medicaments. Carbohydr. Polym..

[162] Yudin V.E., Dobrovolskaya I.P., Neelov I.M., Dresvyanina E.N., Popryadukhin P.V., Ivan'kova E.M., Elokhovskii V.Y., Kasatkin I.A., Okrugin B.M., Morganti P. (2014). Wet spinning of fibers made of chitosan and chitin nanofibrils. Carbohydr. Polym..

[163] Ma B.M., Qin A.W., Li X., Zhao X.Z., He C.J. (2014). Bioinspired design and chitin whisker reinforced chitosan membrane. Mater. Lett..

[164] Ma B.M., Qin A.W., Li X., Zhao X.Z., He C.J. (2014). Structure and properties of chitin whisker reinforced chitosan membranes. Int. J. Biol. Macromol..

[165] Colosi C., Costantini M., Latini R., Ciccarelli S., Stampella A., Barbetta A., Massimi M., Devirgiliis L.C., Dentini M. (2014). Rapid prototyping of chitosan-coated alginate scaffolds through the use of a 3D fiber deposition technique. J. Mater. Chem..

[166] Rubentheren V., Ward T.A., Chee C.Y., Tang C.K. (2015). Processing and analysis of chitosan nanocomposites reinforced with chitin whiskers and tannic acid as a crosslinker. Carbohydr. Polym..

[167] Naseri N., Algan C., Jacobs V., John M., Oksman K., Mathew A.P. (2014). Electrospun chitosan-based nanocomposite mats reinforced with chitin nanocrystals for wound dressing. Carbohydr. Polym..

[168] Tchemtchoua V.T., Atanasova G., Aqil A., Filee P., Garbacki N., Vanhooteghem O., Deroanne C., Noel A., Jerome C., Nusgens B. (2011). Development of a chitosan nanofibrillar scaffold for skin repair and regeneration. Biomacromolecules.

[169] Kim M.S., Park S.J., Gu B.K., Kim C.H. (2012). Polycaprolactone-chitin nanofibrous mats as potential scaffolds for tissue engineering. J. Nanomater..

[170] Ito I., Osaki T., Ifuku S., Saimoto H., Takamori Y., Kurozumi S., Imagawa T., Azuma K., Tsuka T., Okamoto Y. (2014). Evaluation of the effects of chitin nanofibrils on skin function using skin models. Carbohydr. Polym..

[171] Muzzarelli R.A.A. (2009). Genipin-chitosan hydrogels as biomedical and pharmaceutical aids. Carbohydr. Polym..

[172] Charernsriwilaiwat N., Rojanarata T., Ngawhirunpat T., Sukma M., Opanasopit P. (2013). Electrospun chitosan-based nanofiber mats loaded with *Garcinia mangostana* extracts. Int. J. Pharm..

[173] Arslan A., Simsek M., Aldemir S.D., Kazaroglu N.M., Gumusderelioglu M. (2014). Honey-based PET or PET/chitosan fibrous wound dressings: Effect of honey on electrospinning process. J. Biomater. Sci.-Polym. Ed..

[174] Xu J., Cai N., Xu W.X., Xue Y.A., Wang Z.L., Dai Q., Yu F.Q. (2013). Mechanical enhancement of nanofibrous scaffolds through polyelectrolyte complexation. Nanotechnology.

[175] Jridi M., Hajji S., Ben Ayed H., Lassoued I., Mbarek A., Kammoun M., Souissi N., Nasri M. (2014). Physical, structural, antioxidant and antimicrobial properties of gelatin-chitosan composite edible films. Int. J. Biol. Macromol..

[176] Tsai R.Y., Hung S.C., Lai J.Y., Wang D.M., Hsieh H.J. (2014). Electrospun chitosan-gelatin-polyvinyl alcohol hybrid nanofibrous mats: Production and characterization. J. Taiwan Inst. Chem. Eng..

[177] Chen Z.G., Wang P.W., Wei B., Mo X.M., Cui F.Z. (2010). Electrospun collagen-chitosan nanofiber: A biomimetic extracellular matrix for endothelial cell and smooth muscle cell. Acta Biomater..

[178] Wang P.W., Liu J.L., Zhang T. (2013). *In vitro* biocompatibility of electrospun chitosan/collagen scaffold. J. Nanomater..

[179] Sarkar S.D., Farrugia B.L., Dargaville T.R., Dhara S. (2013). Chitosan-collagen scaffolds with nano/microfibrous architecture for skin tissue engineering. J. Biomed. Mater. Res. A.

[180] Dhurai B., Nachimuthu S., Maheswaran, Kumar G., Babu R. (2013). Electrospinning of chitosan nanofibres loaded with curcumin for wound healing. J. Polym. Mater..

[181] Du F.Y., Wang H., Zhao W., Li D., Kong D.L., Yang J., Zhang Y.Y. (2012). Gradient nanofibrous chitosan/poly epsilon-caprolactone scaffolds as extracellular microenvironments for vascular tissue engineering. Biomaterials.

[182] Huang R., Deng H.B., Cai T.J., Zhan Y.F., Wang X.K., Chen X.X., Ji A.L., Li X.Y. (2014). Layer-by-layer immobilized catalase on electrospun nanofibrous mats protects against oxidative stress induced by hydrogen peroxide. J. Biomed. Nanotechnol..

[183] Cai Z.X., Mo X.M., Zhang K.H., Fan L.P., Yin A.L., He C.L., Wang H.S. (2010). Fabrication of chitosan + silk fibroin composite nanofibers for wound-dressing applications. Int. J. Mol. Sci..

[184] Ang-atikarnkul P., Watthanaphanit A., Rujiravanit R. (2014). Fabrication of cellulose nanofiber/chitin whisker/silk sericin bionanocomposite sponges and characterizations of their physical and biological properties. Compos. Sci. Technol..

[185] Zhou Y.S., Yang H.J., Liu X., Mao J., Gu S.J., Xu W.L. (2013). Electrospinning of carboxyethyl chitosan/poly(vinyl alcohol)/silk fibroin nanoparticles for wound dressings. Int. J. Biol. Macromol..

[186] Dunne L.W., Iyyanki T., Hubenak J., Mathur A.B. (2014). Characterization of dielectrophoresis-aligned nanofibrous silk fibroin-chitosan scaffold and its interactions with endothelial cells for tissue engineering applications. Acta Biomater..

[187] Oh B., Lee C.H. (2013). Nanofiber for cardiovascular tissue engineering. Expert Opin. Drug Deliv..

[188] Nawalakhe R., Shi Q., Vitchuli N., Noar J., Caldwell J.M., Breidt F., Bourham M.A., Zhang X., McCord M.G. (2013). Novel atmospheric plasma enhanced chitosan nanofiber/gauze composite wound dressings. J. Appl. Polym. Sci..

[189] Guan Y., Bian J., Peng F., Zhang X.M., Sun R.C. (2014). High strength of hemicelluloses based hydrogels by freeze/thaw technique. Carbohydr. Polym..

[190] Guan Y., Zhang B., Bian J., Peng F., Sun R.C. (2014). Nanoreinforced hemicellulose-based hydrogels prepared by freeze-thaw treatment. Cellulose.

[191] Hatanaka D., Yamamoto K., Kadokawa J. (2014). Preparation of chitin nanofiber-reinforced carboxymethyl cellulose films. Int. J. Biol. Macromol..

[192] Huang Y., Zhang L.N., Yang J., Zhang X.Z., Xu M. (2013). Structure and properties of cellulose films reinforced by chitin whiskers. Macromol. Mater. Eng..

[193] Toskas G., Heinemann S., Heinemann C., Cherif C., Hund R.D., Roussis V., Hanke T. (2012). Ulvan and ulvan/chitosan polyelectrolyte nanofibrous membranes as a potential substrate material for the cultivation of osteoblasts. Carbohydr. Polym..

[194] Tan M.L., Shao P., Friedhuber A.M., van Moorst M., Elahy M, Indumathy S., Dunstan D.E., Wei Y.Z., Dass C.R. (2014). The potential role of free chitosan in bone trauma and bone cancer management. Biomaterials.

[195] Norowski P.A., Mishra S., Adatrow P.C., Haggard W.O., Bumgardner J.D. (2012). Suture pullout strength and *in vitro* fibroblast and RAW 264.7 monocyte biocompatibility of genipin crosslinked nanofibrous chitosan mats for guided tissue regeneration. J. Biomed. Mater. Res. A.

[196] Gentile P., Mattioli-Belmonte M., Chiono V., Ferretti C., Baino F., Tondo-Turo C., Vitale-Brovarone C., Pashkuleva I., Reis R.L., Ciardelli G. (2012). Bioreactive glass/polymer composite scaffold mimiking bone tissue. J. Biomed. Mater. Res. A.

[197] Frohbergh M.E., Katsman A., Botta G.R., Lazarovici P., Schauer C.L., Wegst U.G.K., Lelkes P.I. (2012). Electrospun hydroxyapatite-containing chitosan nanofibers crosslinked with genipin for bone tissue engineering. Biomaterials.

[198] Mahapoka E., Arirachakaran P., Watthanaphanit A., Rujiravanit R., Poolthong S. (2012). Chitosan whiskers from shrimp shells incorporated into dimethacrylate-based dental resin sealant. Dent. Mater. J..

[199] Li X.M., Liu W., Sun L.W., Aifantis K.E., Yu B., Fan Y.B., Feng Q.L., Cui F.Z., Watari F. (2014). Resin composites reinforced by nanoscaled fibers or tubes for dental regeneration. BioMed Res. Int..

[200] Dutta A.K., Egusa M., Kaminaka H., Izawa H., Morimoto M., Saimoto H., Ifuku S. (2014). Facile preparation of surface *N*-halamine chitin nanofiber to endow antibacterial and antifungal activities. Carbohydr. Polym..

[201] Shin H.K., Park M., Chung Y.S., Kim H.Y., Jin F.L., Park S.J. (2014). Antimicrobial characteristics of *N*-halaminated chitosan salt/cotton knit composites. J. Ind. Eng. Chem..

[202] Kangwansupamonkon W., Tiewtrakoonwat W., Supaphol P., Kiatkamjornwong S. (2014). Surface modification of electrospun chitosan nanofibrous mats for antibacterial activity. J. Appl. Polym. Sci..

[203] Mi X., Vijayaragavan K.S., Heldt C.L. (2014). Virus adsorption of water-stable quaternized chitosan nanofibers. Carbohydr. Res..

[204] Xu F., Weng B., Gilkerson R., Materon L.A., Lozano K. (2015). Development of tannic acid/chitosan/pullulan composite nanofibers from aqueous solution for potential applications as wound dressing. Carbohydr. Polym..

[205] Zhao Y., Park R.D., Muzzarelli R.A.A. (2010). Chitin deacetylases: Properties and applications. Mar. Drugs.

[206] Zhang H.C., Fang J.Y., Deng Y., Zhao Y.Y. (2014). Optimized production of *Serratia marcescens* B742 mutants for preparing chitin from shrimp shells powders. Int. J. Biol. Macromol..

[207] Zhang H.N., Tachikawa H., Gao X.D., Nakanishi H. (2014). Applied usage of yeast spores as chitosan beads. Appl. Environ. Microbiol..

[208] Berger L.R.R., Stamford T.C.M., Stamford-Arnaud T.M., de Alcantara S.R.C., da Silva A.C., da Silva A.M., do Nascimento A.E., de Campos-Takaki G.M. (2014). Green conversion of agroindustrial wastes into chitin and chitosan by *Rhizopus arrhizus* and *Cunninghamella elegans* strains. Int. J. Mol. Sci..

[209] Chantarasataporn P., Yoksan R., Visessanguan W., Chirachanchai S. (2013). Water-based nano-sized chitin and chitosan as seafood additive through a case study of Pacific white shrimp (*Litopenaeus vannamei*). Food Hydrocoll..

[210] Xu Y.M., Bajaj M., Schneider R., Grage S.L., Ulrich A.S., Winter J., Gallert C. (2013). Transformation of the matrix structure of shrimp shells during bacterial deproteination and demineralization. Microb. Cell Factories.

